# Exercise Interventions for Depression, Anxiety, and Quality of Life in Older Adults With Cancer: A Systematic Review and Meta-Analysis

**DOI:** 10.1001/jamanetworkopen.2024.57859

**Published:** 2025-02-04

**Authors:** Rou Yi Soong, Chen Ee Low, Vanessa Ong, Isaac Sim, Charmaine Lee, Fattah Lee, Lucas Chew, Chun En Yau, Ainsley Ryan Yan Bin Lee, Matthew Zhixuan Chen

**Affiliations:** 1 Yong Loo Lin School of Medicine, National University of Singapore, Singapore; 2 Division of Geriatric Medicine, Department of Medicine, National University Hospital, Singapore

## Abstract

**Importance:**

Cancer and its treatment negatively impact the mental health of older adults. The potential of exercise interventions as a complementary treatment to alleviate the psychological impacts of cancer is promising, but there are gaps in the current literature.

**Objective:**

To determine if exercise interventions are associated with improvements in psychological outcomes among older adults with cancer.

**Data Sources:**

PubMed, Embase, PsycINFO, and Cochrane databases were searched from database inception to November 5, 2024. Search terms used were *geriatrics*, *cancer*, *depression*, *anxiety*, *quality of life*, and *exercise interventions*.

**Study Selection:**

English-language randomized clinical trials (RCTs) that analyzed the association of various exercise interventions with at least 1 of 3 psychological outcomes (depression, anxiety, or health-related quality-of-life [HRQOL]) were included. The control groups were given usual care. Studies were included if the mean age of participants was older than 60 years and had participants with a diagnosis of any cancer regardless of comorbidities.

**Data Extraction and Synthesis:**

Studies were screened, and data were extracted by 2 independent authors. Random-effects meta-analyses and meta-regressions were used for analysis. The Preferred Reporting Items for Systematic Reviews and Meta-Analyses guideline was followed.

**Main Outcomes and Measures:**

The primary outcomes were depression, anxiety, and HRQOL. Standardized mean difference (SMD) was used to quantify the association of exercise interventions with outcomes.

**Results:**

A total of 27 RCTs with 1929 participants were included. Meta-analyses observed an association of exercise with a significant reduction in levels of depression (SMD = −0.53; 95% CI, −0.79 to −0.28) and anxiety (SMD = −0.39; 95% CI, −0.66 to −0.12) and improvements in overall HRQOL (SMD = 0.63; 95% CI, 0.10 to 1.17). Subgroup analyses revealed that mind-body exercise interventions were significantly associated with improved depression (SMD = −0.89; 95% CI, −1.51 to −0.27) and anxiety levels (SMD = −0.77; 95% CI, −1.54 to −0.01) compared with conventional exercise interventions.

**Conclusion:**

In this systematic review and meta-analysis of 27 RCTs, exercise interventions were found to be associated with significantly reduced levels of depression and anxiety and significantly improved HRQOL in older adults with cancer. These findings suggest that health care professionals and policymakers should focus more on implementing exercise interventions to improve mental health outcomes in this vulnerable population.

## Key Points

**Question:**

Is exercise therapy associated with improved depression, anxiety, and health-related quality of life (HRQOL) in older adults with cancer?

**Findings:**

In this systematic review and meta-analysis of 27 randomized clinical trials with 1929 participants, exercise therapy was associated with reduced depression and anxiety severity and improved HRQOL in older adults with cancer. In particular, mind-body exercises (eg, tai chi, yoga, or qigong) improved outcomes significantly.

**Meaning:**

This systematic review meta-analysis found that exercise therapy was associated with reduced depression and anxiety severity and improved HRQOL in older adults with cancer, suggesting that health care professionals and policymakers should focus more on implementing exercise interventions to improve mental health outcomes in this vulnerable population.

## Teaser

This systematic review and meta-analysis analyzes associations of exercise with anxiety, depression, and quality of life from randomized clinical trials of older adults with cancer.

## Introduction

Cancer is a global health challenge, exhibiting an estimated 19.96 million new cases worldwide in 2022, with projections indicating a surge to more than 35 million cases in 2050.^1^ Cancer is associated with age,^2^ and two-thirds of newly-diagnosed cases are made up of adults aged 60 years and older.^3,4^ Adverse effects of cancer manifest both physically and psychologically,^5^ with 30% to 35% of patients having received a psychiatric disorder diagnosis.^6^ Sequelae of cancer include uncertainty about survival,^7^ bodily deterioration,^8^ treatment-induced adverse effects,^9^ phobias, and psychological distress.^10^ Furthermore, cancer treatment often results in many long-term negative outcomes.^11,12,13^ The unfavorable psychiatric milieu attributed to cancer elevates suicide risk by 4.4 times compared with the general population.^14^ The increased distress not only impairs immune surveillance of tumors, but also persistently activates the hypothalamic-pituitary-adrenal axis that regulates stress response,^15^ thereby increasing the risk of recurrence and mortality.^16^ Older adults with comorbidities,^17^ frailty, and lower physiologic reserve^18^ are more susceptible to complications of the disease and treatment.^19^ Thus, it is pertinent to address the psychological impact of cancer to improve quality of life and patient outcomes in this population.

Exercise therapy, defined as activities exceeding routine physical function, positively impacts the well-being of patients with cancer. Andersen et al^20^ and Assi et al^21^ found that exercise therapy alleviates cancer symptoms like depression, anxiety, nausea, fatigue, and sleep disorders, as well as chemotherapy adverse effects, thereby improving quality of life.^22^ Exercise also slows down tumor growth, inflammation, and angiogenesis, while speeding up tumor regression.^23^ Reducing disease burden and recurrence may mitigate the severity of cancer-associated symptoms, indirectly improving the mental well-being of patients with cancer.

Currently, methods of managing poor mental health include pharmacological treatment and cognitive behavioral therapies. However, conventional pharmacological therapies are hindered by potential adverse effects and drug-drug interactions. Especially in older adults, polypharmacy and compromised organ function^18,19^ pose added challenges in drug administration and lower the overall efficacy of pharmacotherapies.^24^ Although studies have proven cognitive behavioral therapy to be effective, patients face pervasive social stigma obtaining mental health support^25^ and may be reluctant to share their feelings with health care professionals.^26^ Thus, exercise therapy emerges as both a preventive and complementary treatment for both cancer and mental health concerns, functioning synergistically with conventional therapies.

Randomized clinical trials (RCT) remain the benchmark in providing the strongest scientific evidence.^27^ In this meta-analysis of RCTs, we assessed the significance of exercise therapy in alleviating depression and anxiety severity and improving quality of life in older patients with cancer. Furthermore, we compared the effects of mind-body and conventional exercises on the aforementioned parameters, a direction not taken before, to our knowledge. By filling this gap in literature, our study aims to provide valuable insight into the potential of exercise therapy as a complementary and synergistic cancer treatment that improves psychological outcomes.

## Methods

### Protocol and Guidance

This systematic review and meta-analysis is reported according to the Preferred Reporting Items for Systematic Reviews and Meta-Analyses (PRISMA) reporting guideline. Our protocol is registered on PROSPERO (CRD42023394964).

### Data Sources, Search Strategy and Definitions

We conducted a literature search in the PubMed, Embase, PsycINFO, and Cochrane databases from database inception to November 5, 2024. Search terms used were *geriatrics*, *cancer*, *depression*, *anxiety*, *quality of life*, and *exercise interventions*. We included relevant synonyms to expand our search for titles, abstracts, and keywords in those databases. Full strategies for the databases are available in eTable 1 in Supplement 1.

### Study Selection: Inclusion and Exclusion Criteria

**Figure 1.**
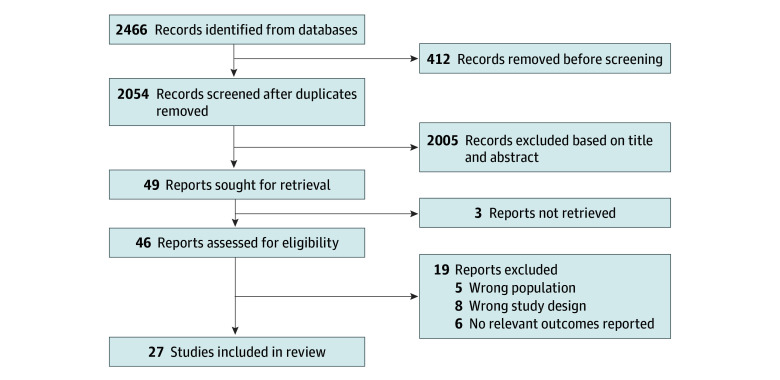
Flowchart of Studies

We included English-language peer-reviewed studies that assessed the association of exercise interventions with psychological outcomes in older adults with cancer. We defined exercise interventions as conventional exercises like aerobic-, resistance-, and strength-related physical training or mind-body exercises such as qigong, yoga, and tai chi, which combine physical movement with mental focus. We included studies if the mean age of patients was 60 years or older,^28^ and participants had a diagnosis of any cancer regardless of comorbidities. We selected only RCTs with control groups (usual care) analyzing at least 1 of these psychological outcomes: depression, anxiety, or health-related quality of life (HRQOL). Studies involving only educational, pharmacological, or surgical interventions were excluded. This selection process is shown in Figure 1.

Based on the aforementioned criteria, 2 independent reviewers (R.Y.S. and V.O.) screened titles and abstracts of all studies. The full text of studies that were assessed to be either relevant or unclear were then reviewed again. Discrepancies were resolved by a third independent reviewer (C.E.L.).

### Data Extraction and Organization

Two reviewers (R.Y.S. and V.O.) independently conducted the extraction process. The data extracted encompassed the study’s objectives, characteristics of the study population, type of exercise intervention and control conducted, alongside primary findings. Standardized mean difference (SMD) was used to quantify the association of exercise interventions. The changes in both mean and SD of depression, anxiety, and HRQOL scores between baseline and postintervention measures were extracted. In the primary analysis, we selected the model from each study that exhibited the highest level of control over confounders. Change in SD was calculated in accordance with Cochrane recommendations; 0.2 represents a small association, 0.5 represents a moderate association, and 0.8 represents a large association.^29^

### Risk of Bias Assessment

We used the Cochrane Risk of Bias Tool 2 checklist to assess risk of bias and methodological quality of the studies. This assessment was done by 2 independent reviewers (R.Y.S. and V.O.).

### Statistical Analysis

Data analyses were conducted using the meta and metafor packages in R version 4.1.0 (R Project for Statistical Computing). Statistical significance was determined by a 2-sided P < .05, unless specified otherwise. SMD was used to aggregate the studies involved in this meta-analysis. Sensitivity analyses were conducted, using leave-one-out analysis, common effects, and identifying and excluding potential outliers. Degree of heterogeneity among studies was evaluated through *I*^2^ and τ^2^ statistics. *I*^2^ less than 30% indicated minimal heterogeneity, 30% to 60% suggested moderate heterogeneity, and *I*^2^ greater than 60% denoted substantial heterogeneity. The potential influence of categorical and hierarchical variables on the results were investigated using subgroup analyses and meta-regression. Quantitative and qualitative assessment of publication bias were done via an Egger test and visual inspection of funnel plot asymmetry, respectively. Because publication bias was suspected, a sensitivity analysis utilizing the trim-and-fill method (R0 estimator, fixed-random effects models) was conducted; this reestimated the overall association size after imputing potentially missing studies, assuming normal distribution of association size around the center of the funnel plot in absence of publication bias.

## Results

Of the 2466 articles identified from databases and citation searching, 2439 studies were omitted after removing duplicates and excluding irrelevant studies. The remaining 27 RCTs^30,31,32,33,34,35,36,37,38,39,40,41,42,43,44,45,46,47,48,49,50,51,52,53,54,55,56^ met the final inclusion criteria (Figure 1).

Of the 27 RCTs, all but one^40^ reported on HRQOL, while 12 studies ^32,35,38,40,44,46,47,50,51,53,55,56^ included data on depression and 9 studies^38,40,44,47,50,51,53,55,56^ included data on anxiety. In total, 1929 participants were included in our analysis, with sample sizes of each study varying from 17 to 252 participants. The majority of studies included patients with prostate cancer (15 studies),^30,32,33,35,36,37,38,40,42,44,45,48,52,54,55^ while 4 others recruited participants with various cancer types,^43,50,51,53^ 3 focused on lung cancer,^39,41,49^ 2 focused on breast cancer,^34,46^ and 1 study each focused on head and neck,^31^ colorectal^47^ and bladder^56^ cancer. A summary of the characteristics of the studies is presented in the Table.

**Table.** Characteristics of Included Studies

Source	Country	Cancer type	Participants, No. (Sex, No. [%])	Race, No. (%)	Age, mean (SD), y	Study methodology and characteristics	Instrument, scales, and diagnostic criteria for assessing depression, anxiety, and HRQOL
Segal et al,^30^ 2003	Canada	Prostate	155 (155 male [100%])	NR	68.0 (7.7)	RCT with males recruited from Ottawa Regional Cancer Centre in Ontario) and Cross Cancer Institute in Edmonton in Alberta	HRQOL: FACT-P
McNeely et al,^31^ 2004	Canada	Head and neck	17 (14 Male [82%]; 3 female [18%])	NR	61.0 (7.7)	RCT with participants recruited from Cross Cancer Institute and University of Alberta in Edmonton	HRQOL: FACT-G
Monga et al,^32^ 2007	US	Prostate	21 (21 Male [100%])	Black (57%); Hispanic (10%); White (33%) (self-reported)	69.2 (4.8)	RCT with males referred for radiotherapy service at the Houston Veterans Affairs Medical Center in Texas for radiation treatment of localized prostate cancer	Depression: BDI; HRQOL: FACT-P
Segal et al,^33^ 2009	Canada	Prostate	121 (121 Male [100%])	NR	66.3 (7.0)	RCT with participants receiving radiation therapy, recruited from Ottawa Hospital Regional Cancer Centre	HRQOL: FACT-G
Banasik et al,^34^ 2010	US	Breast	18 (18 Female [100%])	18 White (100%) (self-reported)	62.9 (6.9)	RCT with females recruited via mail from a local cancer center database in Spokane, Washington	HRQOL: FACT-B
Culos-Reed et al,^35^ 2010	Canada	Prostate	100 (100 Male [100%])	NR	67.6 (8.6)	RCT with participants receiving androgen deprivation therapy recruited from hospitals	Depression: CES-D; HRQOL: EORTC QLQ-C30
Galvão et al,^36^ 2010	Australia	Prostate	57 (57 Male [100%])	NR	69.8 (7.2)	RCT with participants undergoing androgen suppression therapy, recruited from Sir Charles Gairdner Hospital (Perth, Western Australia)	HRQOL: SF-36
Bourke et al,^37^ 2011	UK	Prostate	50 (50 Male [100%])	NR	71.8 (7.0)	RCT with males recruited from outpatient urology clinics in Sheffield	HRQOL: FACT-G
Cormie et al,^38^ 2013	Australia	Prostate	20 (20 Male [100%])	NR	72.2 (7.1)	RCT with participants recruited via referral by oncologists and urologists in Perth, Western Australia	Depression: BSI-18 DEPR; Anxiety: BSI-18 ANX; HRQOL: SF-36
Arbane et al,^39^ 2014	UK	Lung	131 (83 Male [64%]; 48 female [36%])	NR	68.0 (11.0)	RCT with participants admitted for curative surgery, recruited from 2 clinical-academic centers in London	HRQOL: SF-36
Campo et al,^40^ 2014	US	Prostate	40 (40 Male [100%])	3 Other (7%); 37 White (93%) (source of classification NR)^a^	73.8 (8.1)	RCT with males recruited via Huntsman Cancer Institute clinics, University of Utah, cancer registries, and community-based strategies	Depression: BSI-18 DEPR; Anxiety: BSI-18 ANX
Edvardsen et al,^41^ 2014	Norway	Lung	61 (29 Male [47%]; 32 female [53%])	NR	65.2 (8.9)	RCT with participants recruited from Oslo University Hospital	HRQOL: SF-36
Galvão et al,^42^ 2014	Australia and New Zealand	Prostate	100 (100 Male [100%])	NR	71.7 (6.4)	RCT with males enrolled in the RADAR trial (examining effect of adjuvant androgen deprivation therapy duration on recurrence-free survival) from 3 centers, contacted by a letter of invitation from their oncologist	HRQOL: SF-36
Miki et al,^43^ 2014	Japan	Various	78 (35 Male [45%]; 43 female [55%])	NR	74.2 (5.8)	RCT with participants recruited from the outpatient clinic of Hiroshima University Hospital	HRQOL: FACT-G
Cormie et al,^44^ 2015	Australia	Prostate	63 (63 Male [100%])	NR	68.4 (7.1)	RCT with males referred by oncologists and urologists in Perth, Western Australia	Depression: BSI-18 DEPR; Anxiety: BSI-18 ANX; HRQOL: SF-36
Nilsen et al,^45^ 2015	Norway	Prostate	58 (58 Male [100%])	NR	66.0 (5.8)	RCT with males recruited from 2 units at Oslo University Hospital	HRQOL: EORTC QLQ-C30
Yagli et al,^46^ 2015	Turkey	Breast	20 (20 Female [100%])	NR	68.7 (4.7)	RCT with females who were part of the preventive/conservative rehabilitation at the Hacettepe University, Faculty of Health Sciences, Department of Physiotherapy and Rehabilitation, Samanpazari in Ankara	Depression: BDI; HRQOL: NHP
Cramer et al,^47^ 2016	Germany	Colorectal	54 (33 Male [61%]; 21 female [39%])	NR	68.3 (9.7)	RCT with participants recruited from the Department of Surgery and Centre for Minimal Invasive Surgery, Kliniken Essen-Mitte in Essen and the Tempelhof Colon Centre, St. Joseph’s Hospital in Berlin	Depression: HADS; Anxiety: HADS; HRQOL: FACT-C
Winters-Stone et al,^48^ 2016	US	Prostate	64 (64 Male [100%])	59 White (92%); 5 other (8%) (self-reported)^a^	71.8 (7.2)	RCT with participants recruited through the Oregon State Cancer Registry program run by the Oregon Department of Human Service	HRQOL: SF-36
Lai et al,^49^ 2017	China	Lung	60 (34 Male [57%]; 26 female [43%])	NR	72.1 (2.8)	RCT with participants recruited from Department of Thoracic Surgery, West China Hospital, Sichuan University, Chengdu	HRQOL: EORTC-QLQ-C30
Loh et al,^50^ 2019	US	Various	252 (20 Male [8%]; 232 female [92%])	229 White (91%); 23 other (9%) (source of classification NR)^a^	66.7 (5.4)	RCT with participants recruited from 19 community oncology practices across the US	Depression: POMS; Anxiety: STAI; HRQOL: FACT-G emotional well-being scale
Cheng et al,^51^ 2021	China	Various	60 (34 Male [57%]; 26 female [43%])	NR	66.3 (7.6)	RCT with participants recruited from Nanjing Jiangning Hospital and Jiangsu Cancer Hospital	Depression: PHQ-9; Anxiety: GAD-7; HRQOL: QLQ-CCC
Mardani et al,^52^ 2021	Iran	Prostate	80 (80 Male [100%])	NR	69.9 (5.6)	RCT with participants admitted to the radiotherapy department of a large referral teaching hospital in an urban area	HRQOL: EORTC-QLQ-C30
Mikkelsen et al,^53^ 2022	Denmark	Various	84 (36 Male [43%]; 48 female [57%])	NR	71.7 (5.3)	RCT with participants recruited from the Department of Oncology at Copenhagen University Hospital, Herlev, and Gentofte Hospital	Depression: HADS; Anxiety: HADS; HRQOL: EORTC-QLQ-C30
Capela et al,^54^ 2023	Portugal	Prostate	50 (50 Male [100%])	NR	71.8 (5.9)	RCT with participants recruited at the Oncology and Urology departments of the Vila Nova de Gaia-Espinho Hospital Centre	HRQOL: EORTC QLQ-C30
Langlais et al,^55^ 2023	US	Prostate	25 (25 Male [100%])	19 White (76%); 6 other (24%) (source of classification NR)^a^	69.3 (8.4)	RCT with participants living within a 3-hour drive of the University of California, San Francisco, recruited through physician referral and patient lists	Depression: CES-D; Anxiety: STAI; HRQOL: FACT-G
Porserud et al,^56^ 2024	Sweden	Bladder	90 (59 Male [66%]; 31 female [34%])	NR	71.5 (8.5)	RCT with participants recruited from Karolinska University Hospital	Depression: HADS; Anxiety: HADS; HRQOL: EORTC QLQ-C30

Abbreviations: BDI, Beck Depression Inventory; BSI-18-ANX, The Brief Symptom Inventory for Anxiety; BSI-18-DEPR, The Brief Symptom Inventory for Depression; CES-D, Center for Epidemiologic Studies Depression Scale; EORTC-QLQ-C30, European Organisation for the Research and Treatment of Cancer Quality-of-life Questionnaire; FACT-B, The Functional Assessment of Cancer Therapy-Breast; FACT-C, The Functional Assessment of Cancer Therapy-Colorectal; FACT-G, The Functional Assessment of Cancer Therapy-General; FACT-P, The Functional Assessment of Cancer Therapy-Prostate; GAD-7, General Anxiety Disorder-7; HADS, Hospital Anxiety and Depression Scale; HRQOL, health-related quality of life; NHP, Nottingham Health Profile; NR, not reported; PHQ-9, Patient Health Questionnaire; POMS, Profile of Mood States; QLQ-CCC, Quality of Life Questionnaire for Chinese Cancer Patients Receiving Chemobiotherapy; RADAR, Risk-Adapted therapy Directed According to Response; RCT, randomized clinical trial; SF-36, Medical Outcomes Study 36-Item Short Form Health Survey; STAI, State Trait Anxiety Inventory.

^a^ No definition provided for other race.

Various scales were adopted to measure depression, anxiety, and HRQOL. Notably, the Functional Assessment of Cancer Therapy was the most used to measure HRQOL, while the Hospital Anxiety and Depression Scale and Brief Symptom Inventory were most frequently employed to evaluate both depression and anxiety. Exercise interventions varied in frequency, duration, setting, and format. Exercise intensity also varied; some interventions progressively increased the difficulty of exercises at various intervals,^30,31,33,36,38,40,41,42,44,45,47,48,50,52,53,55^ while others kept intensity constant throughout the intervention.^32,34,35,37,39,43,46,49,51,54,56^ Additionally, different interventions were adopted; the majority^30,31,32,33,35,36,37,38,39,41,42,43,44,45,48,49,50,52,54,55,56^ involved conventional exercises, which include structured physical activities like resistance training, aerobic exercise, and strength training and focus primarily on improving physical fitness. The remaining studies^34,40,46,47,51,53^ employed mind-body exercises, such as qigong, yoga, and tai chi, which combine physical movement with mental focus to promote physical, emotional, and mental well-being. All except 4 interventions^39,49,50,55^ took place under the guidance of physiotherapists, instructors, or researchers. Six interventions were conducted in a group setting,^34,36,46,48,51,54^ 12 were conducted individually,^30,31,32,33,37,39,43,45,49,50,55,56^ and the remaining 9 were multimodal, combining both group and individual components.^35,38,40,41,42,44,47,52,53^ All the studies included components conducted in either a hospital or community setting, except for 1 study^50^ that was entirely home-based. Regarding control type, 11 studies^30,32,33,35,41,44,45,50,51,53,55^ assigned their control group to receive only medical therapy, 6 studies^34,36,38,43,47,48^ instructed their control participants to maintain their daily activities, 3 studies^37,52,54^ provided routine check-ups, 2 studies^39,49^ prescribed respiratory care management, 2 studies ^31,40^ prescribed light stretches, 2 studies^46,56^ prescribed other types of exercise, and 1 study^42^ provided an educational booklet. Detailed descriptions of all exercise and control interventions are found in eTable 2 in Supplement 1.

### Significant Improvement in Mean Depression Levels

**Figure 2.**
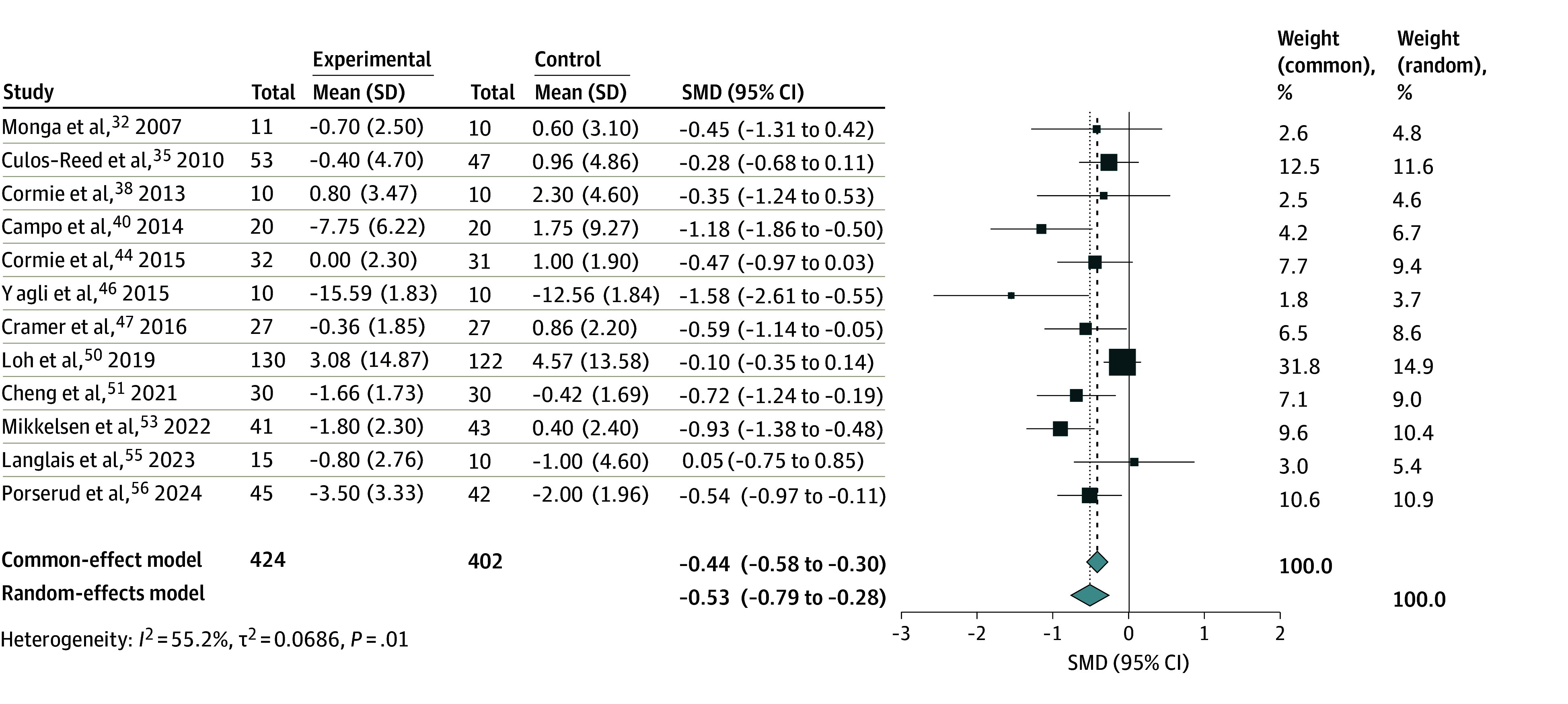
Meta-Analysis of Exercise and Depression Levels Among Older Adults With Cancer The size of the boxes indicates the weight of each study to the overall pooled estimate. SMD indicates standardized mean difference.

Pooled results based on 12 studies^32,35,38,40,44,46,47,50,51,53,55,56^ and 826 participants in a meta-analysis (Figure 2) revealed significant reduction in depression levels after patients underwent exercise programs (SMD = −0.53; 95% CI, −0.79 to −0.28). Results of subgroup analyses of depression severity are presented in eTable 3 in Supplement 1. When the Hospital Anxiety and Depression Scale was used to measure depression, it was associated with a significant decrease in severity of depression (SMD = −0.69; 95% CI, −1.23 to −0.15) compared with other scales. Additionally, mind-body exercises were associated with a more significant reduction in depression severity (SMD = −0.89; 95%CI, −1.51 to −0.27) compared with conventional forms of exercises like aerobic and resistance exercises (SMD = −0.39; 95% CI, −0.64 to −0.13) (eFigure 1 in Supplement 1). However, there was no association of depression levels with other categorical variables such as age, region of study, year of study, type of cancer, control type, or frequency, intensity, and duration of exercise interventions. Meta-regression revealed a significant association of longer exercise interventions (12 weeks or more) with greater reductions in depression severity, compared with shorter interventions. There were no significant associations for age or the effects of exercise intervention over time (eTable 4 in Supplement 1).

### Significant Improvement in Mean Anxiety Levels

**Figure 3.**
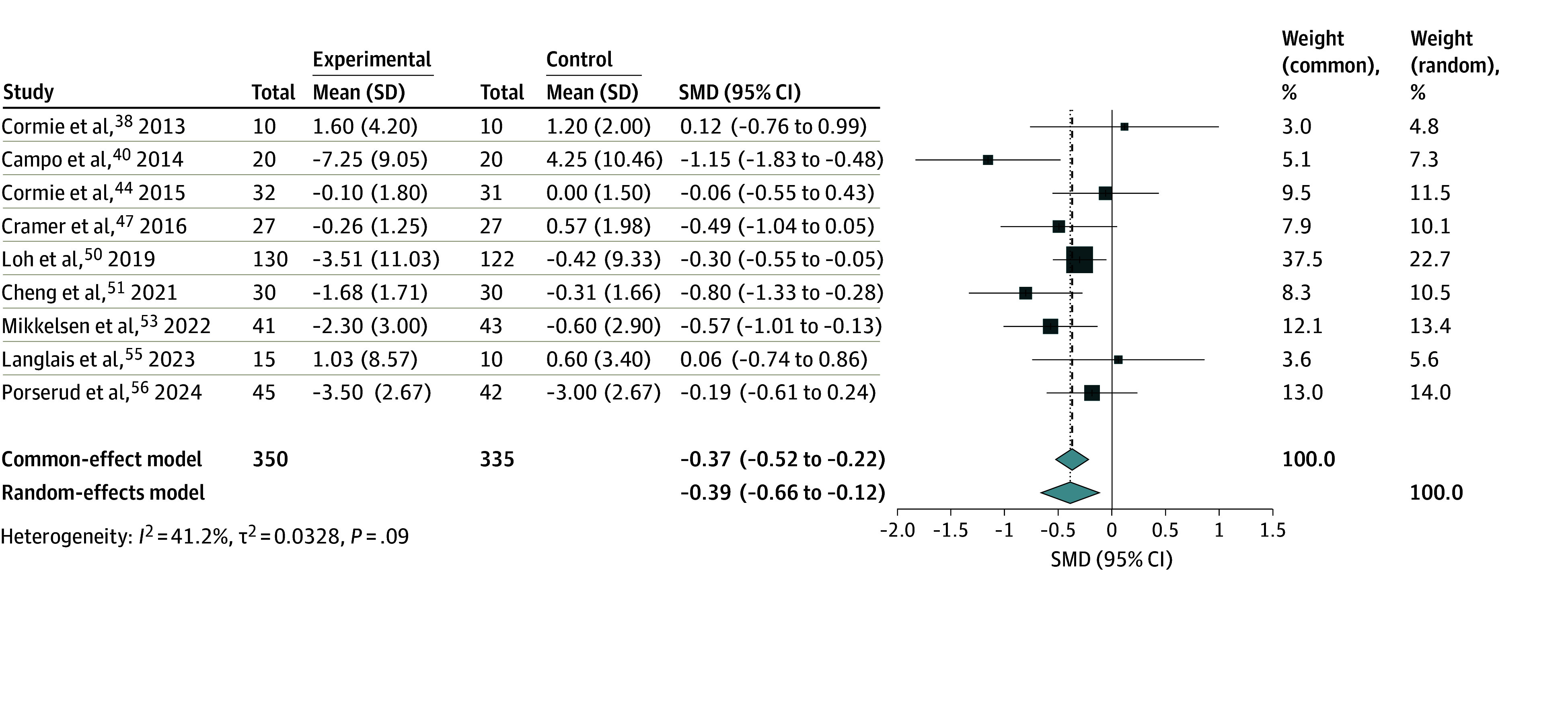
Meta-Analysis of Exercise and Anxiety Levels Among Older Adults With Cancer The size of the boxes indicates the weight of each study to the overall pooled estimate. SMD indicates standardized mean difference.

Meta-analysis of 9 studies^38,40,44,47,50,51,53,55,56^ (Figure 3) was conducted, involving 685 participants. Overall, anxiety levels of participants were significantly reduced after exercise (SMD = −0.39; 95%CI, −0.66 to −0.12). Subgroup analyses of anxiety severity among other categorical variables are listed in eTable 5 in Supplement 1. Mind-body exercises were associated with more significant reductions in anxiety severity (SMD = −0.77; 95% CI, −1.54 to −0.01) compared with conventional exercises (SMD = −0.26; 95% CI, −0.47 to −0.06) (eFigure 1 in Supplement 1). However, there was no association of anxiety levels with other categorical variables such as age, anxiety scale used, region of study, year of study, type of cancer, control type, or frequency and duration of exercise interventions. Meta-regression showed that there was no significant association of exercise interventions with reducing anxiety severity, longer intervention period, and age, or effects of intervention over time (eTable 6 in Supplement 1).

### Significant Improvement in Mean HRQOL Scores

**Figure 4.**
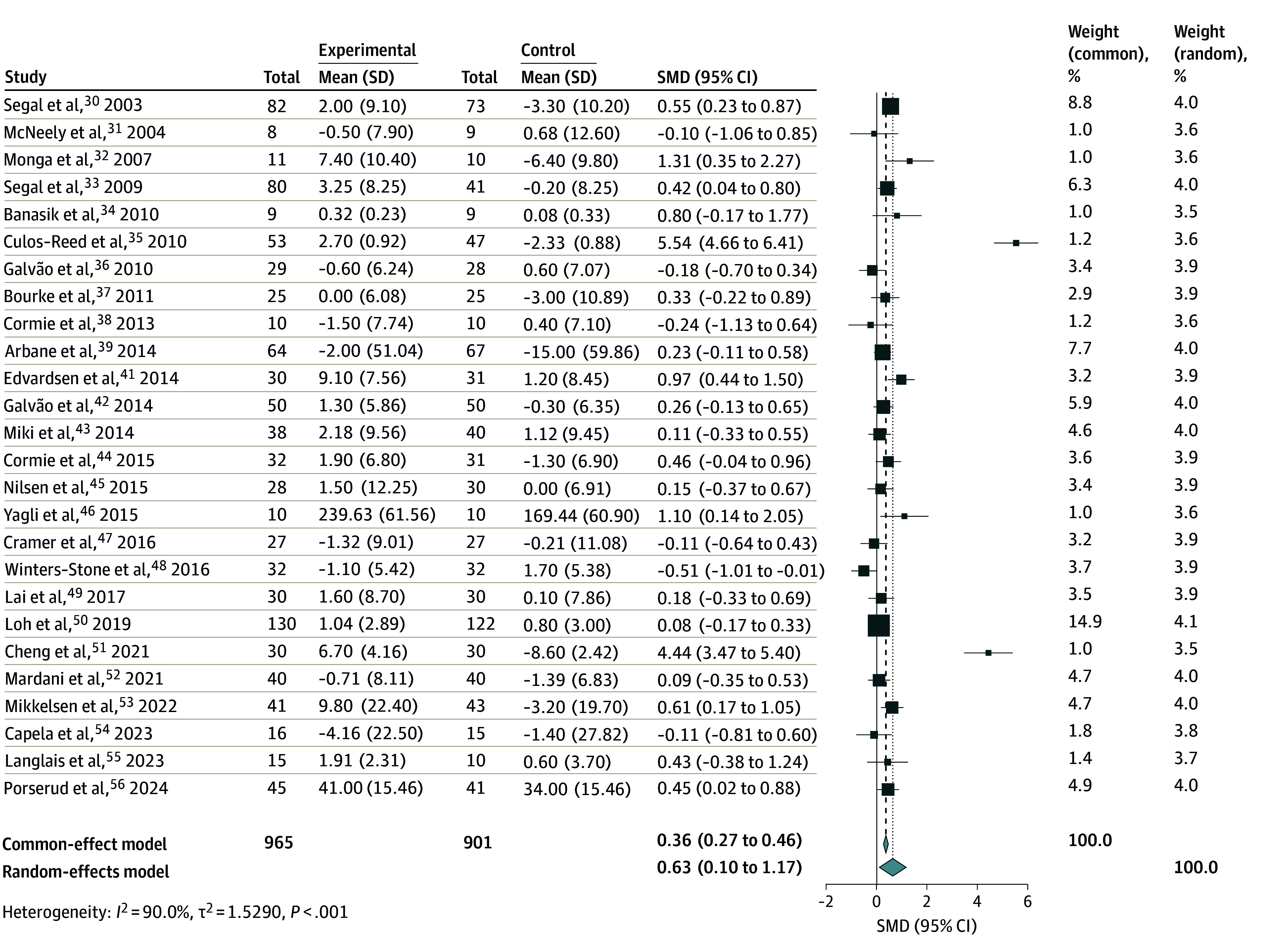
Meta-Analysis of Exercise and Health-Related Quality of Life Levels Among Older Adults With Cancer The size of the boxes indicates the weight of each study to the overall pooled estimate. SMD indicates standardized mean difference.

Across 26 studies,^31,32,33,34,35,36,37,38,39,41,42,43,44,45,46,47,48,49,50,51,52,53,54,55,56^ 1866 participants were included in a meta-analysis to investigate the association of exercise with HRQOL (Figure 4). Overall, HRQOL significantly improved upon implementation of the exercise programs (SMD = 0.63; 95% CI, 0.10-1.17). Subgroup analyses on HRQOL based on other variables are presented in eTable 7 in Supplement 1. When results were stratified based on mean age of participants, the subgroup of participants younger than 70 years demonstrated significantly improved HRQOL levels compared with those with a mean age older than 70 years (SMD = 0.91; 95% CI, 0.11-1.71). However, other categorical variables (type of cancer, type of exercise, control type, region of study, year of study, scale used to measure HRQOL, or frequency, duration, and intensity of intervention) were not associated with a significant improvement in HRQOL scores. Meta-regression showed that there was no significant association of exercise interventions with improving HRQOL, longer intervention period, age, or effects of intervention over time (eTable 8 in Supplement 1).

### Education, Income, Marital Status, Race, and Social Status

Eighteen studies^30,31,32,36,37,38,39,40,42,43,44,46,47,48,49,51,54,56^ examined the association of age with enhancement in psychological well-being of participants after exercising (eTable 9 in Supplement 1). Among these, 17 did not find any significant association of social status with improvement of psychological outcomes. However, 1 study^51^ notably reported that younger males experienced reduced psychological distress compared with older males.

Three studies^40,48,49^ explored the association of race with improvements in psychological outcomes among participants who took part in exercise interventions (eTable 10 in Supplement 1). Eight studies^36,38,40,42,44,47,50,52^ evaluated the association of marital status with psychological outcomes after exercise interventions (eTable 11 in the Supplement). Nine studies^31,36,40,42,43,47,48,52,56^ investigated the association of income level or employment status with improved psychological outcomes after exercise (eTable 12 in Supplement 1). Nine studies^32,36,38,43,44,47,48,50,52^ examined the association of education level with psychological outcomes after exercise (eTable 13 in the Supplement). Nine studies^36,38,39,42,44,49,52,54,56^ investigated the association of smoking status with psychological outcomes after exercising (eTable 14 in the Supplement). Overall, none found a significant association of psychological well-being with race, marital status, income level, employment status, education attainment, or smoking status.

### Risk of Bias, Publication Bias, and Sensitivity Analyses

This meta-analysis assessed the methodological quality of the 27 studies using the Cochrane Risk of Bias Tool 2 (eTable 15 in Supplement 1). Overall, low risk of bias was noted in 10 studies,^31,36,37,39,41,43,44,48,52,56^ potential risk of bias in 15 studies,^30,32,33,34,38,40,42,45,46,47,49,50,51,53,54^ and high risk of bias in 2 studies.^35,55^

In 11 studies,^31,32,34,40,45,46,49,50,51,53,54^ allocation sequence of assignment was not properly concealed, while in 15 studies,^30,33,34,35,37,38,39,42,45,47,49,51,53,55,56^ potential biases arose due to missing outcome data; of those, 2 studies^35,55^ were severely missing data, resulting in a high risk of bias. Overall, 9 studies^32,34,35,36,38,42,46,47,50^ did not implement assessor blinding, possibly giving rise to detection bias. One study^40^ deviated from intended interventions by adopting a per-protocol analysis. As such, the results of the treatment-control analysis should be interpreted after taking the risk of bias analysis into careful consideration. Funnel plots, sensitivity analyses, Egger test, and trim-and-fill procedures indicated the presence of some publication bias (eFigures 2-16 in Supplement 1).

## Discussion

This systematic review and meta-analysis of RCTs demonstrated that exercise was significantly associated with reduction in depression and anxiety severity and enhanced HRQOL among older adults with cancer. Subgroup analyses revealed that mind-body exercises were significantly associated with reduced anxiety and depression levels more than conventional exercises like aerobic or resistance training. To our knowledge, this is the first systematic review and meta-analysis investigating the association of exercise with improving anxiety, depression, and HRQOL in older adults with cancer.

The 2018 American College of Sports Medicine Roundtable found that exercise can improve anxiety, depression, and HRQOL in all cancer survivors.^57^ In our study, exercise interventions were also associated with significant improvement in all psychological outcomes. Reduced severity of depression and anxiety may be due to the following mechanisms. First, exercise exerts antidepressant and anxiolytic effects via the release of neurotransmitters that cross the blood brain barrier to mediate brain-derived neurotrophic factor expression, stimulating hippocampal neurogenesis and improving mood control.^58^ Additionally, exercise stimulates production of monoamines,^59,60^ generating a natural crescendo that stimulates pleasure, satisfaction, and motivation while regulating fatigue.^61^ Furthermore, exercise improves sleep quality,^62^ which has a bidirectional association with depression and anxiety.^63,64^

Mind-body exercises reduce depression and anxiety levels to a significantly greater extent compared with conventional exercises because they focus heavily on relaxation and mindfulness,^65^ potentially stimulating additional mechanisms compared with conventional exercises. Miller et al^66^ found that a walking meditation program resulted in a significantly larger decrease in depression than aerobic walking alone, corroborating our findings and suggesting that additional factors are at play. Conversely, a different study^67^ showed that conventional exercises reduced depression more significantly than mind-body exercises in younger participants; this may be due to differing exercise preferences because older individuals are predisposed to physical limitations and may prefer low-intensity, slower-paced exercises,^68^ while younger individuals may prefer fast-paced, high-intensity exercises.^69^ Additionally, response-shift bias may also affect outcomes because older adults might experience greater mental health benefits from mind-body exercises that align with their preference for mindfulness and relaxation, enhancing perceived effectiveness.^70^ Likewise, younger individuals may perceive greater improvement from exercises that align with their values and lifestyle.^70^

In our analysis, longer interventions, specifically those carried out for at least 12 weeks, were more likely to improve depression scores than those of shorter duration. This finding was expected, as shown by Li et al,^71^ who assessed the effects of different exercise intervention times on depressive symptoms in older adults and found that longer intervention times play a role in ensuring the positive effect of physical exercise. Furthermore, the 2018 American College of Sports Medicine Roundtable found that in particular, thrice-weekly moderate-intensity aerobic training performed for at least 12 weeks significantly reduced anxiety and depression, while an aerobic-resistance program lasting for at least 12 weeks for 2 to 3 times per week improved HRQOL.^57^ Therefore, it is recommended that future exercise interventions be designed for at least 12 weeks.

### Limitations

There are several limitations to this study. We expected heterogenicity in methods of measuring and defining possible important confounding variables like education, income, marital status, and social support, which are well-studied globally^4,19,72^ across different age groups^12,13,73,74^ and cancer types.^10^ We were also unable to control for heterogeneity based on the duration, frequency, and whether the interventions were supervised. Given the variations existing across different study designs and populations, it was unfeasible to statistically pool these variables. As such, we adopted a synthesis without a meta-analysis approach instead. Moreover, because controls did not receive equal amounts of attention as exercise groups, it was not possible to determine how much of the observed effect was due to exercise or simply due to attention.

Next, our study population was intrinsically diverse, with participants undergoing different treatments and varying in their levels of overall health; this introduced variability across the selected studies, which might impact the generalizability of our findings. Moreover, as highlighted by Goel et al,^75^ stressors from factors like disadvantaged neighborhood environments activate the sympathetic nervous system and hypothalamic-pituitary-adrenal axis, which may contribute to more aggressive tumor biology, increasing cancer recurrence and mortality risk. Thus, in addition to the heterogeneity of health and treatment variables in our study, potential effects of geographic or environmental stressors have also not been accounted for. This limitation further underscores the need for future studies to consider the influence of social and environmental contexts on cancer outcomes.

## Conclusions

With advancements in cancer treatment and an aging population, the prevalence of cancer-related decline in psychological well-being can be expected to increase in the following years. Hence, finding effective interventions to mitigate the psychosocial impacts of cancer is essential. Overall, this systematic review and meta-analysis of 27 RCTs found that exercise interventions, especially mind-body exercises, were associated with significant improvements in depression, anxiety, and HRQOL in older adults with cancer. Health care professionals and policymakers should focus more on implementing exercise interventions to improve mental health outcomes in this vulnerable population.

## Notes

Supplement 1. **eTable 1. **Search Strategy **eTable 2. **Specific Details of All Exercise Interventions Used to Improve Psychological Outcomes **eTable 3. **Meta-Analyses of Exercise on Depression Severity in OAC Stratified by Categorical Study-Level Characteristics Using the Random Effect Model **eFigure 1. **Subgroup Meta-Analyses of Exercise on Depression (A) and Anxiety (B) Levels Among Older Adults With Cancer Stratified by Nature of Exercise **eTable 4. **Mixed Effects Meta-Regression of Standardised Mean Differences Against Potential Effect Moderators (Continuous and Categorical Study-Level Characteristics) for Depression Severity After Exercise Interventions in OAC **eTable 5. **Meta-Analyses of Exercise on Anxiety Severity in OAC Stratified by Categorical Study-Level Characteristics Using the Random Effect Model **eTable 6. **Mixed Effects Meta-Regression of Standardised Mean Differences Against Potential Effect Moderators (Continuous and Categorical Study-Level Characteristics) for Anxiety Severity After Exercise Interventions in OAC **eTable 7. **Meta-Analyses of Exercise on HRQOL Improvement in OAC Stratified by Categorical Study-Level Characteristics Using the Random Effect Model **eTable 8. **Mixed Effects Meta-Regression of Standardised Mean Differences Against Potential Effect Moderators (Continuous and Categorical Study-Level Characteristics) for HRQOL Improvement After Exercise Interventions in OAC **eTable 9. **Evaluation of the Mediating or Confounding Effect of Age of Participants on Psychological Outcomes **eTable 10. **Evaluation of the Mediating or Confounding Effect of Race of Participants on Psychological Outcomes **eTable 11. **Evaluation of the Mediating or Confounding Effect of Marital Status of Participants on Psychological Outcomes **eTable 12. **Evaluation of the Mediating or Confounding Effect of Income Level and Employment Status of Participants on Psychological Outcomes **eTable 13. **Evaluation of the Mediating or Confounding Effect of Education Level of Participants on Psychological Outcomes **eTable 14. **Evaluation of the Mediating or Confounding Effect of Smoking Status of Participants on Psychological Outcomes **eTable 15. **Quality Assessment of Included Studies Using the Cochrane Risk-of-Bias Tool 2 **eFigure 2. **Funnel Plot for Visual Inspection of Publication Bias in Studies Assessing Depression Severity in OAC **eFigure 3. **Trim-and-Fill Analysis for Publication Bias in Studies Assessing Depression Severity in OAC **eFigure 4. **Quantitative Assessment Publication Bias in Studies Assessing Depression Severity in OAC **eFigure 5. **Leave-One-Out Analysis of Studies Assessing Depression Severity in OAC, Using the Random Effects Model **eFigure 6. **Outlier Assessment of Studies Assessing Depression Severity in OAC, Using the Random Effects Model **eFigure 7. **Funnel Plot for Visual Inspection of Publication Bias in Studies Assessing Anxiety Severity in OAC **eFigure 8. **Trim-and-Fill Analysis for Publication Bias in Studies Assessing Anxiety Severity in OAC **eFigure 9. **Quantitative Assessment Publication Bias in Studies Assessing Anxiety Severity in OAC **eFigure 10. **Leave-One-Out Analysis of Studies Assessing Anxiety Severity in OAC, Using the Random Effects Model **eFigure 11. **Outlier Assessment of Studies Assessing Anxiety Severity in OAC, Using the Random Effects Model **eFigure 12. **Funnel Plot for Visual Inspection of Publication Bias in Studies Assessing QoL Levels in OAC **eFigure 13. **Trim-and-Fill Analysis for Publication Bias in Studies Assessing QoL Levels in OAC **eFigure 14. **Quantitative Assessment Publication Bias in Studies Assessing QoL Levels in OAC **eFigure 15. **Leave-One-Out Analysis of Studies Assessing QoL Levels in OAC, Using the Random Effects Model **eFigure 16. **Outlier Assessment of Studies Assessing QoL Levels in OAC, Using the Random Effects Model

Supplement 2.
Data Sharing Statement
