# Supplementary material for: Exercise Interventions for Depression, Anxiety, and Quality of Life in Older Adults With Cancer: A Systematic Review and Meta-Analysis
Source: JAMA Netw Open. 2025 Feb 4;8(2):e2457859. doi: 10.1001/jamanetworkopen.2024.57859 (PMC11795328; doi:10.1001/jamanetworkopen.2024.57859)
Supplement: Supplement 1. — eTable 1. Search Strategy eTable 2. Specific Details of All Exercise Interventions Used to Improve Psychological Outcomes eTable 3. Meta-Analyses of Exercise on Depression Severity in OAC Stratified by Categorical Study-Level Characteristics Using the Random Effect Model eFigure 1. Subgroup Meta-Analyses of Exercise on Depression (A) and Anxiety (B) Levels Among Older Adults With Cancer Stratified by Nature of Exercise eTable 4. Mixed Effects Meta-Regression of Standardised Mean Differences Against Potential Effect Moderators (Continuous and Categorical Study-Level Characteristics) for Depression Severity After Exercise Interventions in OAC eTable 5. Meta-Analyses of Exercise on Anxiety Severity in OAC Stratified by Categorical Study-Level Characteristics Using the Random Effect Model eTable 6. Mixed Effects Meta-Regression of Standardised Mean Differences Against Potential Effect Moderators (Continuous and Categorical Study-Level Characteristics) for Anxiety Severity After Exercise Interventions in OAC eTable 7. Meta-Analyses of Exercise on HRQOL Improvement in OAC Stratified by Categorical Study-Level Characteristics Using the Random Effect Model eTable 8. Mixed Effects Meta-Regression of Standardised Mean Differences Against Potential Effect Moderators (Continuous and Categorical Study-Level Characteristics) for HRQOL Improvement After Exercise Interventions in OAC eTable 9. Evaluation of the Mediating or Confounding Effect of Age of Participants on Psychological Outcomes eTable 10. Evaluation of the Mediating or Confounding Effect of Race of Participants on Psychological Outcomes eTable 11. Evaluation of the Mediating or Confounding Effect of Marital Status of Participants on Psychological Outcomes eTable 12. Evaluation of the Mediating or Confounding Effect of Income Level and Employment Status of Participants on Psychological Outcomes eTable 13. Evaluation of the Mediating or Confounding Effect of Education Level of Participants on Psychological Outcomes eTabl [file jamanetwopen-e2457859-s001.pdf]

Supplemental Online Content

Soong RY, Low CE, Ong V, et al. Exercise interventions for depression, anxiety, and quality of life in older adults with cancer: a systematic review and meta-analysis. *JAMA Netw. Open*. 2025;8(2):e2457859. doi:10.1001/jamanetworkopen.2024.57859

eTable 1. Search Strategy
eTable 2. Specific Details of All Exercise Interventions Used to Improve Psychological Outcomes
eTable 3. Meta-Analyses of Exercise on Depression Severity in OAC Stratified by Categorical Study-Level Characteristics Using the Random Effect Model
eFigure 1. Subgroup Meta-Analyses of Exercise on Depression (A) and Anxiety (B) Levels Among Older Adults With Cancer Stratified by Nature of Exercise
eTable 4. Mixed Effects Meta-Regression of Standardised Mean Differences Against Potential Effect Moderators (Continuous and Categorical Study-Level Characteristics) for Depression Severity After Exercise Interventions in OAC
eTable 5. Meta-Analyses of Exercise on Anxiety Severity in OAC Stratified by Categorical Study-Level Characteristics Using the Random Effect Model
eTable 6. Mixed Effects Meta-Regression of Standardised Mean Differences Against Potential Effect Moderators (Continuous and Categorical Study-Level Characteristics) for Anxiety Severity After Exercise Interventions in OAC
eTable 7. Meta-Analyses of Exercise on HRQOL Improvement in OAC Stratified by Categorical Study-Level Characteristics Using the Random Effect Model
eTable 8. Mixed Effects Meta-Regression of Standardised Mean Differences Against Potential Effect Moderators (Continuous and Categorical Study-Level Characteristics) for HRQOL Improvement After Exercise Interventions in OAC
eTable 9. Evaluation of the Mediating or Confounding Effect of Age of Participants on Psychological Outcomes
eTable 10. Evaluation of the Mediating or Confounding Effect of Race of Participants on Psychological Outcomes
eTable 11. Evaluation of the Mediating or Confounding Effect of Marital Status of Participants on Psychological Outcomes
eTable 12. Evaluation of the Mediating or Confounding Effect of Income Level and Employment Status of Participants on Psychological Outcomes
eTable 13. Evaluation of the Mediating or Confounding Effect of Education Level of Participants on Psychological Outcomes
eTable 14. Evaluation of the Mediating or Confounding Effect of Smoking Status of Participants on Psychological Outcomes

© 2025 Soong RY et al. *JAMA Netw. Open*.

eTable 15. Quality Assessment of Included Studies Using the Cochrane Risk-of-Bias Tool 2
eFigure 2. Funnel Plot for Visual Inspection of Publication Bias in Studies Assessing Depression Severity in OAC
eFigure 3. Trim-and-Fill Analysis for Publication Bias in Studies Assessing Depression Severity in OAC
eFigure 4. Quantitative Assessment Publication Bias in Studies Assessing Depression Severity in OAC
eFigure 5. Leave-One-Out Analysis of Studies Assessing Depression Severity in OAC, Using the Random Effects Model
eFigure 6. Outlier Assessment of Studies Assessing Depression Severity in OAC, Using the Random Effects Model
eFigure 7. Funnel Plot for Visual Inspection of Publication Bias in Studies Assessing Anxiety Severity in OAC
eFigure 8. Trim-and-Fill Analysis for Publication Bias in Studies Assessing Anxiety Severity in OAC
eFigure 9. Quantitative Assessment Publication Bias in Studies Assessing Anxiety Severity in OAC
eFigure 10. Leave-One-Out Analysis of Studies Assessing Anxiety Severity in OAC, Using the Random Effects Model
eFigure 11. Outlier Assessment of Studies Assessing Anxiety Severity in OAC, Using the Random Effects Model
eFigure 12. Funnel Plot for Visual Inspection of Publication Bias in Studies Assessing QoL Levels in OAC
eFigure 13. Trim-and-Fill Analysis for Publication Bias in Studies Assessing QoL Levels in OAC
eFigure 14. Quantitative Assessment Publication Bias in Studies Assessing QoL Levels in OAC
eFigure 15. Leave-One-Out Analysis of Studies Assessing QoL Levels in OAC, Using the Random Effects Model
eFigure 16. Outlier Assessment of Studies Assessing QoL Levels in OAC, Using the Random Effects Model

This supplemental material has been provided by the authors to give readers additional information about their work.

© 2025 Soong RY et al. *JAMA Netw. Open*.

eTable 1: Search Strategy

PubMed

#1	"Geriatrics"[Mesh] OR "Aged"[Mesh] OR Aging OR Aged OR Centenarians OR Nonagenarians OR Octogenarians OR Elder* OR Gerontology OR ‘older adult*’
#2	Neoplas*[title/abstract] OR Cancer[title/abstract] OR Malignan*[title/abstract]
#3	Exercis*[title/abstract] OR Exerciz*[title/abstract] OR (Physical*[title/abstract] AND Interven*[title/abstract]) OR (Physical*[title/abstract] AND Activit*[title/abstract])
#4	depress* OR anxi* OR burden* OR Stress* OR ‘quality of life’

Embase

#1	'geriatrics'/exp OR 'aged'/exp OR 'aging'/exp OR 'senescence'/exp OR 'older adult*' OR ‘aging’ OR ‘centenarians’ OR ‘nonagenarians’ OR ‘octogenarians’ OR ‘gerontology’ OR 'older people'/exp
#2	'neoplas*'/exp OR ‘cancer’/exp OR ‘malignan*’/exp
#3	‘exercis*' OR 'exerciz*' OR (physical* NEAR/2 (intervent* OR activit*))
#4	'depress*' OR ‘depression’/exp OR 'anxi*' OR ‘anxiety’/exp OR 'disease burden*' OR 'physiological stress*' OR ‘quality of life’

Cochrane

#1	MeSH descriptor: [Geriatrics] explode all trees OR ((Aging OR Aged OR Centenarians OR Nonagenarians OR Octogenarians OR Elder* OR Gerontology OR ‘older adult*’):ti,ab,kw)
#2	(neoplas*):ti,ab,kw OR (cancer):ti,ab,kw OR (malignan*):ti,ab,kw
#3	MeSH descriptor: [Exercise] explode all trees OR (exercis*' OR 'exerciz*' OR (physical* NEAR/2 (intervent* OR activit*)):ti,ab,kw)
#4	MeSH descriptor: [Depression] explode all trees OR (anxi* OR burden* OR Stress* OR ‘quality of life’):ti,ab,kw)

© 2025 Soong RY et al. *JAMA Netw. Open*.

PsycINFO

#1	(Geriatrics OR Aged OR Aging OR Centenarians OR Nonagenarians OR Octogenarians OR Elder* OR Gerontology OR 'Older Adult*').mp.
#2	(neoplas* OR cancer OR malignan*).ti,ab.
#3	exp Exercise/ OR exercis*.ti,ab. OR exerciz*.ti,ab. OR (physical* adj2 (intervent*.ti,ab. or activit*.ti,ab.))
#4	(depress* OR anxi* OR burden* OR stress* OR ‘quality of life’).ti,ab.

© 2025 Soong RY et al. *JAMA Netw. Open*.

eTable 2: Specific details of all exercise interventions used to improve psychological outcomes

Study	Country	Frequency	Setting	Group-based	Supervision under	Characteristics of intervention	Characteristics of control
Segal et al,[30] 2003	Canada	Thrice a week for 12 weeks	Fitness center	Individual	Instructor	Low-resistance exercise consisted of 9 strength-training exercises, with instructions to increase the resistance when they were able to complete more than 12 repetitions. Patients were free to complete their program at any time during the fitness centers' hours of operation.	Medical therapy consisting of androgen deprivation therapy
McNeely et al,[31] 2004	Canada	Thrice a week for 12 weeks	Cancer center	Individual	Physical therapist	Participants undertook a resistance-training program that was progressive in terms of number of sets and repetitions performed, as well as the amount of weight lifted. The goal of the exercises was to enhance scapular stability and restore or maintain the strength of the upper extremity.	Light passive range-of-movement stretches
Monga et al,[32] 2007	USA	Thrice a week for 8 weeks	Hospital	Individual	Staff kinesio-therapist, physicians	Patients exercised in the morning, before receiving their daily radiotherapy. The exercise protocol consisted of a 10-min warm-up, a 30-min aerobic segment consisting of walking on a treadmill, and a 5- to 10-min cool down period. Patients were instructed to maintain their target heart rate throughout the aerobic component of the program.	Medical therapy consisting of patient advice and radiotherapy
Segal et al,[33] 2009	Canada	Thrice a week for 24 weeks	Rehabilitation center	Individual	Exercise specialists	The first intervention group engaged in a resistance training program involving two sets of 8-12 repetitions of 10 different exercises that progressively increased in resistance. Another intervention group engaged in aerobic training on a cycle ergometer, treadmill, or elliptical trainer that progressively increased in duration and intensity. Warm-up and cool-down periods were 5 mins of light aerobic activity and stretching. Sessions lasted between 15 and 45 mins.	Medical therapy consisting of radiotherapy
Banasik et al,[34] 2010	Canada	Once a week for 4 months	Fitness center	Group and individual	Certified fitness specialists	Participants underwent both a home-based portion and weekly group sessions. Home exercises primarily consisted of walking, stretching, and light resistance exercises. Additionally, participants attended weekly booster sessions in a group-based format conducted in a fitness center, consisting of a 30-mins educational/discussion portion, and a 1h group-based workout similar to their home-based program.	Daily activities post-treatment and not receiving any extra active care
Culos-Reed et al,[35] 2010	Australia	Twice a week for 12 weeks	NR	Group	Exercise physiologist	Participants undertook combined progressive resistance and aerobic training. The resistance exercise program was designed to increase in number of repetitions, while the aerobic component included 15 to 20 mins of cardiovascular exercises (cycling and walking/jogging). Each session lasted approximately 60 mins in total.	Medical therapy consisting of androgen deprivation therapy

© 2025 Soong RY et al. *JAMA Netw. Open*.

Galvao et al,[36] 2010	USA	Twice a week for 8 weeks	NR	Group	Expert Iyengar instructors	Traditional Iyengar yoga routines that progressively increased in difficulty as participants gained strength and flexibility, with each session lasting 90 mins.	Daily activities and not receiving any extra active care
Bourke et al,[37] 2011	UK	Thrice a week for 12 weeks	Exercise suite, community facilities	Individual	Experienced exercise physiologist	Program comprised of 30 mins of aerobic exercise and 2-4 sets of resistance exercises (body weight resistance and free weights) targeting large skeletal muscle groups with an experienced exercise physiologist, twice weekly for the initial 6 weeks and then once weekly for the following 6 weeks. In addition, they were required to undertake self-directed exercise (e.g., brisk walking, cycling, and gym exercise) for at least one 30-min session per week during the initial 6 weeks and at least 2 sessions per week for the final 6 weeks, using a logbook to record activity.	Routine follow-up at the urology clinic
Cormie et al,[38] 2013	Australia	Twice a week for 12 weeks	Exercise clinic, home	Group and individual	Accredited exercise physiologist	Resistance exercise sessions lasting 60 minutes were conducted in groups of 1-5 participants, commencing with a 5-min warm-up period and ending with a 10-min cool-down period consisting of low-level aerobic exercise and stretching. Resistance exercise regime included eight exercises that target the major muscle groups of the upper and lower body, that progressively increased in intensity and volume. Participants were encouraged to supplement with home-based aerobic exercise sessions to accumulate 150 mins of moderate intensity exercise each week.	Daily activities such a dietary and social and usual self-management regimen, and not receiving any extra active care
Arbane et al,[39] 2014	UK	Daily for 4 weeks	Hospital, home	Individual	Unsupervised	For the first 5 days post-operatively, patients received a once-daily cycle and strength training. Following their discharge, they were prescribed a home walking program and were given a pedometer with a daily walking target of 30 continuous minutes. All patients were telephoned weekly and offered general encouragement.	Airway clearance technique and routine chest physiotherapy
Campo et al,[40] 2014	USA	Twice a week for 12 weeks	Center, home	Group and individual	Certified Qigong instructors	60 mins-long Qigong sessions began with a five-minute meditative focus on the breath, followed by sitting exercises, then standing movements, and ended with a final 5-min meditative focus on the breath; as the study progressed, a larger proportion of time was spent performing standing movements. Additionally, participants were provided with a DVD with tutorials by the instructors to encourage home-based practice.	Light stretches that target main upper and lower body muscle groups, performed seated and standing
Edvardsen et al,[41] 2014	Australia	Twice a week for 3 months	Exercise clinic, home	Group and individual	Accredited exercise physiologists	Participants engaged in moderate–high intensity aerobic and resistance exercises. The aerobic exercise component included 20–30 mins of cardiovascular exercise (walking, jogging on a treadmill, cycling etc.) while the 30-min resistance exercise component involved exercises targeting major upper and lower body muscle groups. Intensity of aerobic and resistance exercises were progressive and modified according to individual response. Participants were also encouraged to supplement the supervised exercise	Medical therapy consisting of standard oncological care

© 2025 Soong RY et al. *JAMA Netw. Open*.

						sessions with home-based aerobic exercise to accumulate a total of at least 150 mins of moderate intensity aerobic exercise each week.	
Galvao et al,[42] 2014	Norway	Thrice a week for 20 weeks	Fitness centers	Group and individual	Qualified personal trainers, physiotherapists	The exercise program was individualised and included a cardiovascular warm-up, high-intensity training (walking uphill on a treadmill), progressive resistance training and daily inspiratory muscle training. Each session lasted 60 mins.	Modified educational booklet with a general recommendation to perform 150 min per week of moderate physical activity during the entire 12-months period
Miki et al,[43] 2014	Australia	Four times a week for 6 months	Exercise clinic, home	Group and individual	Exercise physiologist	Participants undertook combined progressive resistance and aerobic training in groups for twice per week. The resistance exercises progressed in intensity and included chest press, seated row, shoulder press, triceps extension, leg press, leg extension and leg curl, and abdominal crunches. The aerobic training component included 20–30 mins of cardiovascular exercises (cycling and walking/jogging). In addition, two more exercise sessions were completed at home each week involving aerobic exercise.	Daily activities and not receiving any extra active care
Cormie et al,[44] 2015	Japan	Once a week for 4 weeks	Hospital	Individual	Therapists	Participants pedalled the ergometer while visually tracking their path displaced on the PC screen. Exercise load and the maximal number of rotations were set at 20 W and 80 rpm respectively. Participants pedalled for 5 mins.	Medical therapy consisting of androgen deprivation therapy
Nilsen et al,[45] 2015	Norway	Thrice a week for 16 weeks	Training facility	Individual	Instructor	Participants underwent a high-load strength training including nine exercises, which progressively increased in training volume and duration.	Medical therapy consisting of androgen deprivation therapy
Yagli et al,[46] 2015	Turkey	Once a week for 8 weeks	Hospital	Group	Certified and experienced Yoga teachers	The program included 15 mins warm-up and breathing exercises, 15 mins of Yoga poses and 30 mins of relaxation and meditation in supine position.	1h physical exercises, once a week for 8 weeks
Cramer et al,[47] 2016	Germany	Once a week for 10 weeks	Center, home	Group and individual	Certified and experienced Hatha Yoga instructors	Each 90-min class started with low-intensity warm-up exercises, followed by a series of yoga postures. Postures taught increased in difficulty and intensity during the program. Each class then ended with yogic breathing techniques. Patients were also encouraged to indicate their daily home yoga practice into a daily log, although no minimal practice time was required.	Daily activities post-surgery and not receiving any extra active care

© 2025 Soong RY et al. *JAMA Netw. Open*.

Winters-Stone et al,[48] 2016	USA	Twice a week for 6 months	University center	Group	Exercise physiologist	Participants underwent a progressive strength training program with their spouses. Within a 60-minutes session, participants performed 8–10 different exercises, evenly split between upper and lower body training. A 5-min dynamic aerobic warm-up and 5–10 min stretching cool-down were performed at the beginning and end of each exercise session.	Daily activities post-treatment such as usual physical activity habits and not receiving any extra active care
Lai et al,[49] 2017	China	Daily for 7 days	Rehabilitation training center	Individual	Not reported	Alongside abdominal breathing training and expiration exercise, patients underwent daily 30-min aerobic endurance training with a NuStep device (cross trainer machine), where they adjusted the resistance gear range according to their own speed and power.	Conventional pre-operative respiratory management
Loh et al,[50] 2019	USA	Daily for 6 weeks	Home	Individual	Unsupervised	EXCAP is a home-based, progressive, low- to moderate-intensity aerobic and resistance exercise program. Participants were provided with an exercise kit containing a pedometer, 3 therapeutic bands and an instruction manual). For the aerobic component, participants wore the pedometer and recorded their daily steps; for the resistance component, they performed exercises with the therapeutic bands. Participants were encouraged to progressively increase their steps and intensity of resistance band exercises each week.	Medical therapy consisting of chemotherapy
Cheng et al,[51] 2021	China	Thrice a week for 12 weeks	NR	Group	Experienced Tai Chi instructor	The 24-form simplified Yang-style Tai Chi was performed repeatedly for a total of 30 mins. The instructor explained and demonstrated the moves, which was then followed by the subjects. There was a 5-min warm-up exercise before the exercise and 5-min cool-down exercise after training.	Medical therapy consisting of chemotherapy or radiotherapy
Mardani et al,[52] 2021	Iran	4 times a week for 12 weeks	Urban park	Group and individual	Researcher	The exercise program included aerobic, resistant and flexible exercises, with incremental increases in walking time and intensity of weigh-tolerance and resistance exercises. Participants took part in 1 session of assigned group exercise at the urban park and three sessions of individual exercise per week using exercise facilities in the community. Each session lasted 60 to 150 mins.	Routine check-ups
Mikkelsen et al,[53] 2022	Denmark	Twice a week for 12 weeks	Hospital, home	Group and individual	Physiotherapists	60-min long group-based progressive resistance training consisting of seven exercises targeting all major muscle groups. If participants were prevented from attending an exercise session, they received a replacement training program with a few effective exercises that could be performed at home. Additionally, participants were also provided with a pedometer for a home individualized walking program to help maintain or increase level of daily activity.	Medical therapy consisting of standard oncological treatment
Capela et al,[54] 2023	Portugal	Thrice a week for 16 weeks	Indoor sports hall	Group	Exercise physiologists	Walking football program divided into four sequential phases: 15-min warm-up phase involving joint mobilityand balance exercises; 50-min skill-developing phase (football-specific technical skills and fundamental motor	Routine follow-up appointments with attending physician, regular screenings and

© 2025 Soong RY et al. *JAMA Netw. Open*.

						skills like aerobic power or muscular endurance); 20-min structured small, sided game (e.g., 7 vs. 7 or 5 vs. 5); and 5-min cool-down phase.	general counselling on issues related to physical inactivity and weight gain
Langlais et al,[55] 2023	USA	Thrice a week for 12 weeks	Exercise facility	Individual	Unsupervised	The first intervention group engaged in aerobic exercise sessions using a cycle ergometer; the program was mostly vigorous, interspersed with moderate exercise to balance out exertion levels. Another intervention group engaged in resistance exercise arm consisting of 8 exercises for the upper and lower body, progressively increasing in number of sets and repetitions across sessions. Participants were unsupervised, but could reach out to gym staff or trainers for help if needed.	Medical therapy consisting of androgen deprivation therapy
Porserud et al,[56] 2024	Sweden	Twice a week for 12 weeks	Primary care, community facilities	Individual	Physiotherapist	The program consisted of aerobic exercise aiming for moderate intensity (30 min/session) and strengthening exercises for endurance training with 2×15 repetitions. Patients were also recommended to take daily walks with recommended number of daily steps set together with physiotherapist every week.	Unsupervised home-based exercise, given written and oral instructions for a gradually increasing exercise program including daily walks and a sit-to-stand exercise

© 2025 Soong RY et al. *JAMA Netw. Open*.

eTable 3: Meta-analyses of exercise interventions on depression severity in OAC stratified by categorical study-level characteristics using the random effect model

Variable	Cohorts	N, intervention	N, control	SMD	95% CI	I2	Test of interaction (p-value)
Overall	12	424	402	-0.53	-0.79; -0.28	55%	NA
Prostate Cancer	6	141	128	-0.45	-0.86; -0.04	25%	0.21
Breast Cancer	1	10	10	-1.58	-2.61; -0.55	NA	
Colorectal Cancer	1	27	27	-0.59	-1.14; -0.05	NA	
Bladder Cancer	1	45	42	-0.54	-0.97; -0.11	NA	
Various Cancers	3	201	195	-0.53	-1.64; 0.58	83%	
Age<70	8	308	287	-0.40	-0.71; -0.09	47%	0.08
Age >70	4	116	115	-0.77	-1.29; -0.25	22%	
PHQ-9	1	30	30	-0.72	-1.24; -0.19	NA	**0.03**
POMS-D	1	130	122	-0.10	-0.35; 0.14	NA	
HADS-D	3	113	112	-0.69	-1.23; -0.15	0%	
BSI-18	3	62	61	-0.66	-1.71; 0.39	39%	
BDI	2	21	20	-0.92	-8.05; 6.20	63%	
CES-D	2	68	57	-0.20	-2.03; 1.62	0%	
America	5	229	209	-0.31	-0.82; 0.20	57%	0.06
Europe	3	113	112	-0.69	-1.22; -0.16	0%	
East Asia	1	30	30	-0.72	-1.24; -0.19	NA	
Australia	2	42	41	-0.43	-1.10; 0.23	0%	
Middle East	1	10	10	-1.58	-2.61; -0.55	NA	
Mind-body exercises	4	87	87	-0.89	-1.51; -0.27	24%	**0.02**

© 2025 Soong RY et al. *JAMA Netw. Open*.

Traditional aerobic or resistance exercises	8	337	315	-0.39	-0.64; -0.13	42%	
Thrice a week	3	56	50	-0.44	-1.42; 0.53	20%	0.20
Twice a week	5	148	146	-0.70	-1.09; -0.31	18%	
Once a week	3	90	84	-0.57	-1.85; 0.72	64%	
Daily	1	130	122	-0.10	-0.35; 0.14	NA	
12 weeks or more	8	246	233	-0.57	-0.87; -0.28	34%	0.69
Less than 12 weeks	4	178	169	-0.46	-1.30; 0.38	68%	
Progressive increments	7	275	263	-0.50	-0.89; -0.11	66%	0.70
No progressive increments	5	149	139	-0.60	-1.09; -0.10	34%	
Study published before 2011	2	64	57	-0.33	-1.29; 0.63	0%	0.27
Study published from 2011-2020	6	229	220	-0.57	-1.10; -0.04	69%	
Study published after 2020	4	131	125	-0.60	-1.19; -0.01	37%	
Control group assigned to medical therapy	7	312	293	-0.41	-0.73; -0.10	55%	0.34
Control group assigned to daily activities	2	37	37	-0.51	-1.96; 0.94	0%	
Control group assigned to stretches	1	20	20	-1.18	-1.86; -0.50	NA	
Control group assigned to other exercise types	2	55	52	-0.80	-6.54; 4.94	70%	

Abbreviations: PHQ-9, Patient Health Questionnaire; POMS-D, Profile of Mood States Depression Scale; HADS-D, Hospital Anxiety and Depression Scale; BSI-18, The Brief Symptom Inventory; BDI, Beck Depression Inventory; SMD, standardized mean difference; NA, not applicable; CI, confidence interval

© 2025 Soong RY et al. *JAMA Netw. Open*.

eTable 4: Mixed effects meta-regression of standardised mean differences against potential effect moderators (continuous and categorical study-level characteristics) for depression severity after exercise interventions in OAC

	Ratio	P	95% CI Lower	95% CI Upper	I^2 (% residual heterogeneity)
Age over 70 years old	-0.1682	0.5990	-1.3372	1.0009	71.92%
Age between 60 and 70 years old	0.0498	0.8631	-0.6272	0.7268	33.85%
Duration of intervention over 12 weeks	-0.3805	**0.0311**	-0.7150	-0.0460	46.02%
Duration of intervention under 12 weeks	-0.0595	0.7929	-0.9145	0.7956	58.59%
Year of publication between 2011 and 2020	0.0088	0.9500	-0.3580	0.3757	73.42%
Year of publication after 2020	0.2155	0.1106	-0.1214	0.5524	0.00%

Abbreviations: CI, confidence interval

© 2025 Soong RY et al. *JAMA Netw. Open*.

eTable 5: Meta-analyses of exercise interventions on anxiety severity in OAC stratified by categorical study-level characteristics using the random effect model

Variable	Cohorts	N, intervention	N, control	SMD	95% CI	I2	Test of interaction (p-value)
Overall	9	350	335	-0.39	-0.66; -0.12	41%	NA
Prostate Cancer	4	77	71	-0.28	-1.23; 0.67	65%	0.79
Colorectal Cancer	1	27	27	-0.49	-1.04; 0.05	NA	
Bladder Cancer	1	45	42	-0.19	-0.61; 0.24	NA	
Various Cancers	3	201	195	-0.53	-1.15; 0.10	40%	
Age<70	5	234	220	-0.34	-0.73; 0.04	28%	0.66
Age >70	4	116	115	-0.46	-1.22; 0.29	60%	
GAD-7	1	30	30	-0.80	-1.33; -0.28	NA	0.49
STAI	2	145	132	-0.20	-2.25; 1.85	0%	
HADS-A	3	113	112	-0.41	-0.93; 0.11	0%	
BSI-18	3	62	61	-0.37	-2.03; 1.28	75%	
America	3	165	152	-0.43	-1.66; 0.81	70%	**<0.01**
Europe	3	113	112	-0.41	-0.93; 0.12	0%	
East Asia	1	30	30	-0.80	-1.33; -0.28	NA	
Australia	2	42	41	-0.01	-1.03; 1.02	0%	
Mind-body exercises	3	77	77	-0.77	-1.54; -0.01	11%	**<0.01**
Traditional aerobic or resistance exercises	6	273	258	-0.26	-0.47; -0.06	0%	
Thrice a week	2	45	40	-0.45	-5.83; 4.93	68%	0.99

© 2025 Soong RY et al. *JAMA Netw. Open*.

Twice a week	5	148	146	-0.38	-0.96; 0.20	58%	
Once a week	1	27	27	-0.49	-1.04; 0.05	NA	
Daily	1	130	122	-0.30	-0.55; -0.05	NA	
12 weeks or more	7	193	186	-0.40	-0.81; 0.01	54%	0.62
Less than 12 weeks	2	157	149	-0.37	-1.55; 0.81	0%	
Progressive increments	7	275	263	-0.37	-0.72; -0.02	42%	0.79
No progressive increments	2	75	72	-0.46	-4.35; 3.43	69%	
Study published from 2011-2020	5	219	210	-0.37	-0.88; 0.15	52%	0.85
Study published after 2020	4	131	125	-0.42	-0.98; 0.14	41%	
Control group assigned to medical therapy	5	248	236	-0.37	-0.74; 0.00	36%	0.19
Control group assigned to daily activities	2	37	37	-0.31	-3.89; 3.28	26%	
Control group assigned to stretches	1	20	20	-1.15	-1.83; -0.48	NA	
Control group assigned to other exercise types	2	45	42	-0.19	-0.61; 0.24	NA	

Abbreviations: GAD-7, General Anxiety Disorder-7; STAI, The State Trait Anxiety Inventory; HADS-A, Hospital Anxiety and Depression Scale; BSI-18, The Brief Symptom Inventory; SMD, standardized mean difference; NA, not applicable; CI, confidence interval

© 2025 Soong RY et al. *JAMA Netw. Open*.

eTable 6: Mixed effects meta-regression of standardised mean differences against potential effect moderators (continuous and categorical study-level characteristics) for anxiety severity after exercise interventions in OAC

	Ratio	P	95% CI Lower	95% CI Upper	I^2 (% residual heterogeneity)
Age over 70 years old	-0.1682	0.2717	0.5990	-1.3372	71.92%
Age between 60 and 70 years old	0.2374	0.4380	-0.6093	1.0841	40.90%
Year of publication between 2011 and 2020	-0.0014	0.9899	-0.3325	0.3296	65.24%
Year of publication after 2020	0.2155	0.1106	-0.1214	0.5524	0.00%

Abbreviations: CI, confidence interval

© 2025 Soong RY et al. *JAMA Netw. Open*.

eTable 7: Meta-analyses of exercise interventions on HRQOL improvement in OAC stratified by categorical study-level characteristics using the random effect model

Variable	Cohorts	N, intervention	N, control	SMD	95% CI	I2	Test of interaction (p-value)
Overall	26	965	901	0.63	0.10; 1.17	90%	NA
Prostate Cancer	14	503	442	0.59	-0.25; 1.43	92%	0.65
Breast Cancer	2	19	19	0.95	-0.92; 2.82	0%	
Lung Cancer	3	124	128	0.46	-0.64; 1.56	67%	
Colorectal Cancer	1	27	27	-0.11	-0.64; 0.43	N A	
Head & Neck Cancer	1	8	9	-0.10	-1.06; 0.85	NA	
Bladder Cancer	1	45	41	0.45	0.02; 0.88	NA	
Various Cancers	4	239	235	1.24	-2.02; 4.50	96%	
Age<70	17	678	615	0.91	0.11; 1.71	93%	0.05
Age >70	9	287	286	0.13	-0.15; 0.40	46%	
EORTC QLQ C-30	8	283	276	1.34	-0.48; 3.16	97%	0.38
FACT	10	425	366	0.37	0.07; 0.68	38%	
SF-36	7	247	249	0.15	-0.31; 0.61	70%	
NHP	1	10	10	1.10	0.14; 2.05	NA	
East Asia	3	98	100	1.48	-4.57; 7.53	97%	0.46
America	9	420	353	0.91	-0.45; 2.28	95%	
Europe	8	276	279	0.32	0.02; 0.62	43%	
Australia	4	121	119	0.08	-0.46; 0.62	26%	
Middle East	2	50	50	0.57	-5.83; 6.96	72%	

© 2025 Soong RY et al. *JAMA Netw. Open*.

Mind-body exercises	5	117	119	1.32	-0.86; 3.50	94%	0.31
Traditional aerobic or resistance exercises	21	848	782	0.48	-0.06; 1.02	89%	
Daily	3	224	219	0.16	-0.03; 0.35	0%	0.46
Four times a week	2	90	90	0.18	-0.91; 1.26	0%	
Thrice a week	10	325	274	0.81	-0.12; 1.74	88%	
Twice a week	7	198	194	0.20	-0.27; 0.66	67%	
Once a week	4	128	124	1.60	-2.57; 5.76	98%	
12 weeks or more	18	646	586	0.72	-0.05; 1.49	93%	0.49
Less than 12 weeks	8	319	315	0.44	0.00; 0.87	43%	
Progressive increments	15	634	577	0.20	-0.02; 0.41	56%	0.06
No progressive increments	11	331	324	1.25	0.00; 2.49	95%	
Study published before 2011	7	272	217	1.16	-0.66; 2.97	96%	0.28
Study published from 2011-2020	13	506	505	0.23	-0.03; 0.49	50%	
Study published after 2020	6	187	179	0.94	-0.81; 2.68	93%	
Control group assigned to medical therapy	11	532	468	1.30	0.09; 2.51	95%	0.16
Control group assigned to daily activities	6	145	146	-0.03	-0.50; 0.43	29%	
Control group assigned to stretches	1	8	9	-0.10	-1.06; 0.85	NA	
Control group assigned to other exercise types	2	55	51	0.76	-3.36; 4.87	32%	
Control group assigned to educational intervention	1	50	50	0.26	-0.13; 0.65	NA	
Control group assigned to respiratory care management	2	94	97	0.21	-0.13; 0.54	0%	
Control group assigned to routine check-ups	3	81	80	0.11	-0.44; 0.65	0%	

Abbreviations: EORTC QLQ C-30, European Organization for the Research and Treatment of Cancer Quality of Life Questionnaire; FACT, The Functional Assessment of Cancer Therapy; SF-36, Medical Outcomes Study 36-Item Short Form Health Survey; NHP, Nottingham Health Profile; SMD, standardized mean difference; NA, not applicable; CI, confidence interval

© 2025 Soong RY et al. *JAMA Netw. Open*.

eTable 8: Mixed effects meta-regression of standardised mean differences against potential effect moderators (continuous and categorical study-level characteristics) for HRQOL improvement after exercise interventions in OAC

	Ratio	P	95% CI Lower	95% CI Upper	I^2 (% residual heterogeneity)
Age over 70 years old	0.0272	0.8777	-0.3753	0.4296	52.88%
Age between 60 and 70 years old	0.0350	0.8447	-0.3391	0.4090	97.49%
Duration of intervention over 12 weeks	-0.0746	0.3655	-0.2443	0.0951	96.95%
Duration of intervention under 12 weeks	0.0283	0.6392	-0.1119	0.1685	49.59%
Year of publication before 2011	0.2308	0.4406	-0.4778	0.9395	97.20%
Year of publication between 2011 and 2020	-0.0531	0.3151	-0.1641	0.0579	45.45%
Year of publication after 2020	-0.6847	0.3186	-2.3552	0.9857	96.40%

Abbreviations: CI, confidence interval

© 2025 Soong RY et al. *JAMA Netw. Open*.

eTable 9: Evaluation of the mediating or confounding effect of age of participants on psychological outcomes

Author	Year	Country	Study population	Key findings† Age
Segal et al,[30] 2003	2003	Canada	155 males with prostate cancer, mean age 68.0, SD 7.69 recruited from Ottawa Regional Cancer Center (Ottawa, Ontario, Canada) and Cross Cancer Institute in Edmonton (Alberta, Canada)	There was no significant association found between age and improved psychological outcomes in OAC as compared to control (p=0.66).
McNeely et al,[31] 2004	2004	Canada	17 participants with head and neck cancer, mean age 61, SD 7.7 recruited from Cross Cancer Institute and University of Alberta in Edmonton, Canada	There was no significant association found between age and improved psychological outcomes in OAC as compared to control (p=0.62).
Monga et al,[32] 2007	2007	USA	21 men with prostate cancer referred for radiotherapy service mean age 69.2 years, SD 4.82 recruited from Houston Veterans Affairs Medical Center	There was no significant association found between age and improved psychological outcomes in OAC as compared to control (p=0.23).
Galvao et al,[36] 2009	2009	Australia	57 males with prostate cancer without bone metastases undergoing androgen suppression therapy, mean age 69.8, SD 7.24 recruited from Sir Charles Gairdner Hospital (Perth, Western Australia).	There was no significant association found between age and improved psychological outcomes in OAC as compared to control (p=0.75).
Bourke et al,[37] 2011	2011	UK	50 males with prostate cancer, mean age 71.8, SD 7.02 recruited from outpatient urology clinics in Sheffield	There was no significant association found between age and improved psychological outcomes in OAC as compared to control (p=0.66).
Cormie et al,[38] 2013	2013	Australia	20 men with bone metastatic disease secondary to prostate cancer mean age 72.15 years, SD 7.08 were recruited via referral by oncologists and urologists in Perth, Western Australia	There was no significant association found between age and improved psychological outcomes in OAC as compared to control (p=0.57).
Arbane et al,[39] 2014	2014	UK	131 patients with lung cancer, mean age 68, SD 11 recruited from two clinical–academic centers in London, UK	There was no significant association found between age and improved psychological outcomes in OAC as compared to control (p=0.70).
Campo et al,[40] 2014	2014	USA	40 men with prostate cancer mean age 73.8 years, SD 8.12 recruited via Huntsman Cancer Institute clinics, cancer registries, and community-based strategies	There was no significant association found between age and improved psychological outcomes in OAC as compared to control (p=0.81).
Galvao et al,[42] 2014	2014	Australia	100 males with prostate cancer, mean age 71.7, SD 6.42 recruited from three centers in Australia and New Zealand	There was no significant association found between age and improved psychological outcomes in OAC as compared to control (p>0.05).

© 2025 Soong RY et al. *JAMA Netw. Open*.

Miki et al,[43] 2014	2014	Japan	78 patients with breast or prostate cancer mean age 74.2 years, SD 5.78 recruited from the outpatient clinic of Hiroshima University Hospital	There was no significant association found between age and improved psychological outcomes in OAC as compared to control (p=0.15).
Cormie et al,[44] 2015	2015	Australia	63 males with prostate cancer, mean age 68.4, SD 7.07 referred by oncologists and urologists in Perth, Western Australia	There was no significant association found between age and improved psychological outcomes in OAC as compared to control (p=0.15).
Yagli et al,[46] 2015	2015	Turkey	20 women with breast cancer mean age 68.73 years, SD 4.70 recruited from Hacettepe University, Faculty of Health Sciences, Department of Physiotherapy and Rehabilitation	There was no significant association found between age and improved psychological outcomes in OAC as compared to control (p=0.91).
Cramer et al,[47] 2016	2016	Germany	54 patients with colorectal cancer mean age 68.26 years, SD 9.69, who are 2 to 48 months post-surgery, recruited from the Department of Surgery and Center for Minimal Invasive Surgery, Kliniken Essen-Mitte, Essen, Germany, and the Tempelhof Colon Center, St. Joseph's Hospital, Berlin, Germany	There was no significant association found between age and improved psychological outcomes in OAC as compared to control (p=0.92).
Winters-Stone et al,[48] 2016	2016	USA	64 men with prostate cancer residing with a spouse, mean age 71.75 years, SD 7.24 recruited through the Oregon State Cancer Registry program run by the Oregon Department of Human Service	There was no significant association found between age and improved psychological outcomes in OAC as compared to control (p>0.05).
Lai et al,[49] 2017	2017	China	60 patients with primary NSCLC scheduled to undergo LC lobectomy, mean age 72.05 years, SD 2.77 recruited from Department of Thoracic Surgery, West China Hospital	There was no significant association found between age and improved psychological outcomes in OAC as compared to control (p=0.23).
Cheng et al,[51] 2021	2021	China	60 patients with lung, gastric or breast cancer mean age 66.3 (7.55) experiencing some degree of CRF as indicated by the Brief Fatigue Inventory, recruited from Nanjing Jiangning Hospital and Jiangsu Cancer Hospital	**Young participants reported a greater improvement in psychological outcomes over time compared with the control group (p=0.001).**
Capela et al,[54] 2023	2023	Capela	50 men with prostate cancer, mean age 71.8, SD 5.9 recruited at the Oncology and Urology departments of the Vila Nova de Gaia-Espinho Hospital Center, Portugal	There was no significant association found between age and improved psychological outcomes in OAC as compared to control (p=0.34).
Porserud et al,[56] 2024	2024	Sweden	90 patients with urinary bladder cancer, mean age 71.5 SD 8.5 recruited from Karolinska University Hospital, Sweden	There was no significant association found between age and improved psychological outcomes in OAC as compared to control (p>0.05).

Abbreviations: SD, standard deviation; OAC, older adults with cancer

†Outcomes of interest include logistic or linear regression analysis for any association between level of education attainment and improvement in psychological outcomes (depression, anxiety, HRQOL)

© 2025 Soong RY et al. *JAMA Netw. Open*.

eTable 10: Evaluation of the mediating or confounding effect of race of participants on psychological outcomes

Author	Year	Country	Study population	Key findings† Race
Campo et al,[40] 2014	2014	USA	40 men with prostate cancer mean age 73.8 years, SD 8.12 recruited via Huntsman Cancer Institute clinics, cancer registries, and community-based strategies	There was no significant association found between race and improved psychological outcomes in OAC as compared to control (p=0.62).
Winters-Stone et al,[48] 2016	2016	USA	64 men with prostate cancer residing with a spouse, mean age 71.75 years, SD 7.24 recruited through the Oregon State Cancer Registry program run by the Oregon Department of Human Service	There was no significant association found between race and improved psychological outcomes in OAC as compared to control (p>0.05).
Loh et al,[50] 2019	2019	USA	252 patients with primary diagnosis of cancer other than leukaemia mean age 66.7 years, SD 5.4 recruited from 19 community oncology practices across the United States	There was no significant association found between race and improved psychological outcomes in OAC as compared to control (p=0.62).

Abbreviations: SD, standard deviation; OAC, older adults with cancer

†Outcomes of interest include logistic or linear regression analysis for any association between level of education attainment and improvement in psychological outcomes (depression, anxiety, HRQOL)

© 2025 Soong RY et al. *JAMA Netw. Open*.

eTable 11: Evaluation of the mediating or confounding effect of marital status of participants on psychological outcomes

Author	Year	Country	Study population	Key findings† Marital status
Galvao et al,[36] 2009	2009	Australia	57 males with prostate cancer without bone metastases undergoing androgen suppression therapy, mean age 69.8, SD 7.24 recruited from Sir Charles Gairdner Hospital (Perth, Western Australia).	There was no significant association found between marital status and improved psychological outcomes in OAC as compared to control (p=0.25).
Cormie et al,[38] 2013	2013	Australia	20 men with bone metastatic disease secondary to prostate cancer mean age 72.15 years, SD 7.08 were recruited via referral by oncologists and urologists in Perth, Western Australia	There was no significant association found between marital status and improved psychological outcomes in OAC as compared to control (p=0.53).
Campo et al,[40] 2014	2014	USA	40 men with prostate cancer mean age 73.8 years, SD 8.12 recruited via Huntsman Cancer Institute clinics, cancer registries, and community-based strategies	There was no significant association found between marital status and improved psychological outcomes in OAC as compared to control (p=0.34).
Galvao et al,[42] 2014	2014	Australia	100 males with prostate cancer, mean age 71.7, SD 6.42 recruited from three centers in Australia and New Zealand	There was no significant association found between marital status and improved psychological outcomes in OAC as compared to control (p>0.05).
Cormie et al,[44] 2015	2015	Australia	63 males with prostate cancer, mean age 68.4, SD 7.07 referred by oncologists and urologists in Perth, Western Australia	There was no significant association found between marital status and improved psychological outcomes in OAC as compared to control (p=0.67).
Cramer et al,[47] 2016	2016	Germany	54 patients with colorectal cancer mean age 68.26 years, SD 9.69, who are 2 to 48 months post-surgery, recruited from Kliniken Essen-Mitte Hospital, Essen, Germany, and the Tempelhof Colon Center, St. Joseph’s Hospital, Berlin, Germany	There was no significant association found between marital status and improved psychological outcomes in OAC as compared to control (p=0.40).
Loh et al,[50] 2019	2019	USA	252 patients with primary diagnosis of cancer other than leukaemia mean age 66.7 years, SD 5.4 recruited from 19 community oncology practices across the United States	There was no significant association found between marital status and improved psychological outcomes in OAC as compared to control (p=0.67).
Mardani et al,[52] 2021	2021	Iran	80 men with prostate cancer admitted for radiotherapy mean age 69.9 years, SD 5.59 recruited from a large referral teaching hospital in an urban area of Iran	There was no significant association found between marital status and improved psychological outcomes in OAC as compared to control (p>0.05).

Abbreviations: SD, standard deviation; OAC, older adults with cancer

†Outcomes of interest include logistic or linear regression analysis for any association between level of education attainment and improvement in psychological outcomes (depression, anxiety, HRQOL)

© 2025 Soong RY et al. *JAMA Netw. Open*.

eTable 12: Evaluation of the mediating or confounding effect of income level and employment status of participants on psychological outcomes

Author	Year	Country	Study population	Key findings† Income level & employment status
McNeely et al,[31] 2004	2004	Canada	17 participants with head and neck cancer, mean age 61, SD 7.7 recruited from Cross Cancer Institute and University of Alberta in Edmonton, Canada	There was no significant association found between employment status and improved psychological outcomes in OAC as compared to control (p=0.99).
Galvao et al,[36] 2009	2009	Australia	57 males with prostate cancer without bone metastases undergoing androgen suppression therapy, mean age 69.8, SD 7.24 recruited from Sir Charles Gairdner Hospital (Perth, Western Australia).	There was no significant association found between employment status and improved psychological outcomes in OAC as compared to control (p=0.41).
Campo et al,[40] 2014	2014	USA	40 men with prostate cancer mean age 73.8 years, SD 8.12 recruited via Huntsman Cancer Institute clinics, cancer registries, and community-based strategies	There was no significant association found between employment status (p=0.67) or income level (p=1.00) and improved psychological outcomes in OAC as compared to control.
Galvao et al,[42] 2014	2014	Australia	100 males with prostate cancer, mean age 71.7, SD 6.42 recruited from three centers in Australia and New Zealand	There was no significant association found between employment status and improved psychological outcomes in OAC as compared to control (p>0.05).
Miki et al,[43] 2014	2014	Japan	78 patients with breast or prostate cancer mean age 74.2 years, SD 5.78 recruited from the outpatient clinic of Hiroshima University Hospital	There was no significant association found between employment status and improved psychological outcomes in OAC as compared to control (p=0.51).
Cramer et al,[47] 2016	2016	Germany	54 patients with colorectal cancer mean age 68.26 years, SD 9.69, who are 2 to 48 months post-surgery, recruited from the Department of Surgery and Center for Minimal Invasive Surgery, Kliniken Essen-Mitte, Essen, Germany, and the Tempelhof Colon Center, St. Joseph's Hospital, Berlin, Germany	There was no significant association found between employment status and improved psychological outcomes in OAC as compared to control (p=0.87).
Winters-Stone et al,[48] 2016	2016	USA	64 men with prostate cancer residing with a spouse, mean age 71.75 years, SD 7.24 recruited through the Oregon State Cancer Registry program run by the Oregon Department of Human Service	There was no significant association found between employment status and improved psychological outcomes in OAC as compared to control (p>0.05).

© 2025 Soong RY et al. *JAMA Netw. Open*.

Mardani et al,[52] 2021	2021	Iran	80 men with prostate cancer admitted for radiotherapy mean age 69.9 years, SD 5.59 recruited from a large referral teaching hospital in an urban area of Iran	There was no significant association found between employment or economic status and improved psychological outcomes in OAC as compared to control (p>0.05).
Porserud et al,[56] 2024	2024	Sweden	90 patients with urinary bladder cancer, mean age 71.5 SD 8.5 recruited from Karolinska University Hospital, Sweden	There was no significant association found between employment status and improved psychological outcomes in OAC as compared to control (p>0.05).

Abbreviations: SD, standard deviation; OAC, older adults with cancer

†Outcomes of interest include logistic or linear regression analysis for any association between level of education attainment and improvement in psychological outcomes (depression, anxiety, HRQOL)

© 2025 Soong RY et al. *JAMA Netw. Open*.

eTable 13: Evaluation of the mediating or confounding effect of education level of participants on psychological outcomes

Author	Year	Country	Study population	Key findings†<br>Education level
Monga et al,[32] 2007	2007	USA	21 men with prostate cancer referred for radiotherapy service mean age 69.2 years, SD 4.82 recruited from Houston Veterans Affairs Medical Center	There was no significant association found between education level and improved psychological outcomes in OAC as compared to control (p=0.44)
Galvao et al,[36] 2009	2009	Australia	57 males with prostate cancer without bone metastases undergoing androgen suppression therapy, mean age 69.8, SD 7.24 recruited from Sir Charles Gairdner Hospital (Perth, Western Australia).	There was no significant association found between having post-secondary education and improved psychological outcomes in OAC as compared to control (p=0.13).
Cormie et al,[38] 2013	2013	Australia	20 men with bone metastatic disease secondary to prostate cancer mean age 72.15 years, SD 7.08 were recruited via referral by oncologists and urologists in Perth, Western Australia	There was no significant association found between having a tertiary education and improved psychological outcomes in OAC as compared to control (p=1.00).
Miki et al,[43] 2014	2014	Japan	78 patients with breast or prostate cancer mean age 74.2 years, SD 5.78 recruited from the outpatient clinic of Hiroshima University Hospital	There was no significant association found between years of education and improved psychological outcomes in OAC as compared to control (p=0.54).
Cormie et al,[44] 2015	2015	Australia	63 males with prostate cancer, mean age 68.4, SD 7.07 referred by oncologists and urologists in Perth, Western Australia	There was no significant association found between having tertiary education and improved psychological outcomes in OAC as compared to control (p=0.29).
Cramer et al,[47] 2016	2016	Germany	54 patients with colorectal cancer mean age 68.26 years, SD 9.69, who are 2 to 48 months post-surgery, recruited from the Department of Surgery and Center for Minimal Invasive Surgery, Kliniken Essen-Mitte, Essen, Germany, and the Tempelhof Colon Center, St. Joseph's Hospital, Berlin, Germany	There was no significant association found between education level and improved psychological outcomes in OAC as compared to control (p=0.54)
Winters-Stone et al,[48] 2016	2016	USA	64 men with prostate cancer residing with a spouse, mean age 71.75 years, SD 7.24 recruited through the Oregon State Cancer Registry program run by the Oregon Department of Human Service	There was no significant association found between having above high school education and improved psychological outcomes in OAC as compared to control (p>0.05).

© 2025 Soong RY et al. *JAMA Netw. Open*.

Loh et al,[50] 2019	2019	USA	252 patients with primary diagnosis of cancer other than leukaemia mean age 66.7 years, SD 5.4 recruited from 19 community oncology practices across the United States	There was no significant association found between education level and improved psychological outcomes in OAC as compared to control (p=0.70)
Mardani et al,[52] 2021	2021	Iran	80 men with prostate cancer admitted for radiotherapy mean age 69.9 years, SD 5.59 recruited from a large referral teaching hospital in an urban area of Iran	There was no significant association found between having a diploma education or higher and psychological outcomes in OAC as compared to control (p>0.05).

Abbreviations: SD, standard deviation; OAC, older adults with cancer

†Outcomes of interest include logistic or linear regression analysis for any association between level of education attainment and improvement in psychological outcomes (depression, anxiety, HRQOL)

© 2025 Soong RY et al. *JAMA Netw. Open*.

eTable 14: Evaluation of the mediating or confounding effect of smoking status of participants on psychological outcomes

**Author**	**Year**	**Country**	**Study population**	**Key findings†**<br>**Smoking status**
Galvao et al,[36] 2009	2009	Australia	57 males with prostate cancer without bone metastases undergoing androgen suppression therapy, mean age 69.8, SD 7.24 recruited from Sir Charles Gairdner Hospital (Perth, Western Australia).	There was no significant association found between current smoking status and improved psychological outcomes in OAC as compared to control (p=0.96)
Cormie et al,[38] 2013	2013	Australia	20 men with bone metastatic disease secondary to prostate cancer mean age 72.15 years, SD 7.08 were recruited via referral by oncologists and urologists in Perth, Western Australia	There was no significant association found between current smoking status and improved psychological outcomes in OAC as compared to control (p=1.00)
Arbane et al,[39] 2014	2014	UK	131 patients with lung cancer, mean age 68, SD 11 recruited from two clinical–academic centers in London, UK	There was no significant association found between pack years and improved psychological outcomes in OAC as compared to control (p=0.64).
Galvao et al,[42] 2014	2014	Australia	100 males with prostate cancer, mean age 71.7, SD 6.42 recruited from three centers in Australia and New Zealand	There was no significant association found between current smoking status and improved psychological outcomes in OAC as compared to control (p>0.05)
Cormie et al,[44] 2015	2015	Australia	63 males with prostate cancer, mean age 68.4, SD 7.07 referred by oncologists and urologists in Perth, Western Australia	There was no significant association found between current (p=0.96) or past (0.99) smoking status and improved psychological outcomes in OAC as compared to control.
Lai et al,[49] 2017	2017	China	60 patients with primary NSCLC scheduled to undergo LC lobectomy, mean age 72.05 years, SD 2.77 recruited from Department of Thoracic Surgery, West China Hospital	There was no significant association found between current smoking status and improved psychological outcomes in OAC as compared to control (p=0.74)
Mardani et al,[52] 2021	2021	Iran	80 men with prostate cancer admitted for radiotherapy mean age 69.9 years, SD 5.59 recruited from a large referral teaching hospital in an urban area of Iran	There was no significant association found between current smoking status and improved psychological outcomes in OAC as compared to control (p>0.05)

© 2025 Soong RY et al. *JAMA Netw. Open*.

Capela et al,[54] 2023	2023	Capela	50 men with prostate cancer, mean age 71.8, SD 5.9 recruited at the Oncology and Urology departments of the Vila Nova de Gaia-Espinho Hospital Center, Portugal	There was no significant association found between current smoking status and improved psychological outcomes in OAC as compared to control (p=1.00)
Porserud et al,[56] 2024	2024	Sweden	90 patients with urinary bladder cancer, mean age 71.5 SD 8.5 recruited from Karolinska University Hospital, Sweden	There was no significant association found between current smoking status and improved psychological outcomes in OAC as compared to control (p>0.05)

Abbreviations: SD, standard deviation; OAC, older adults with cancer

†Outcomes of interest include logistic or linear regression analysis for any association between level of education attainment and improvement in psychological outcomes (depression, anxiety, HRQOL)

© 2025 Soong RY et al. *JAMA Netw. Open*.

eTable 15: Quality assessment of included studies using the Cochrane risk-of-bias tool 2.

Study	D1	D2	D3	D4	D5	Overall
	Risk of bias domains					
Segal et al, 2003 [30]	+	+	X	+	+	-
McNeely et al, 2004 [31]	-	+	+	+	+	+
Monga et al, 2007 [32]	-	+	+	-	+	-
Segal et al, 2009 [33]	+	+	-	+	+	-
Banasik et al, 2010 [34]	-	+	-	-	+	-
Culos-Reed et al, 2010 [35]	+	+	X	-	+	X
Galvao et al, 2010 [36]	+	+	+	-	+	+
Bourke et al, 2011 [37]	+	+	-	+	+	+
Cormie et al, 2013 [38]	+	+	-	-	+	-
Arbane et al, 2014 [39]	+	+	-	+	+	+
Campo et al, 2014 [40]	-	-	+	+	+	-
Edvardsen et al, 2014 [41]	+	+	+	+	+	+
Galvao et al, 2014 [42]	+	+	-	-	+	-
Miki et al, 2014 [43]	+	+	+	+	+	+
Cormie et al, 2015 [44]	+	+	+	+	+	+
Nilsen et al, 2015 [45]	-	+	-	+	+	-
Yagli et al, 2015 [46]	-	+	+	-	+	-
Cramer et al, 2016 [47]	+	+	-	-	+	-
Winters-Stone et al, 2016 [48]	+	+	+	+	+	+
Lai et al, 2017 [49]	-	+	-	+	+	-
Loh et al, 2019 [50]	-	+	+	-	+	-
Cheng et al, 2021 [51]	-	+	-	+	+	-
Mardani et al, 2021 [52]	+	+	+	+	+	+
Mikkelsen et al, 2022 [53]	-	+	-	+	+	-
Capela et al, 2023 [54]	-	+	+	+	+	-
Langlais et al, 2023 [55]	+	+	X	+	+	X
Porserud et al, 2024 [56]	+	+	-	+	+	+

Domains:
D1: Bias arising from the randomization process.
D2: Bias due to deviations from intended intervention.
D3: Bias due to missing outcome data.
D4: Bias in measurement of the outcome.
D5: Bias in selection of the reported result.

Judgement
X High
- Some concerns
+ Low

© 2025 Soong RY et al. *JAMA Netw. Open*.

eFigure 1: Subgroup meta-analyses of exercise on depression (A) and anxiety (B) levels among older adults with cancer stratified by nature of exercise

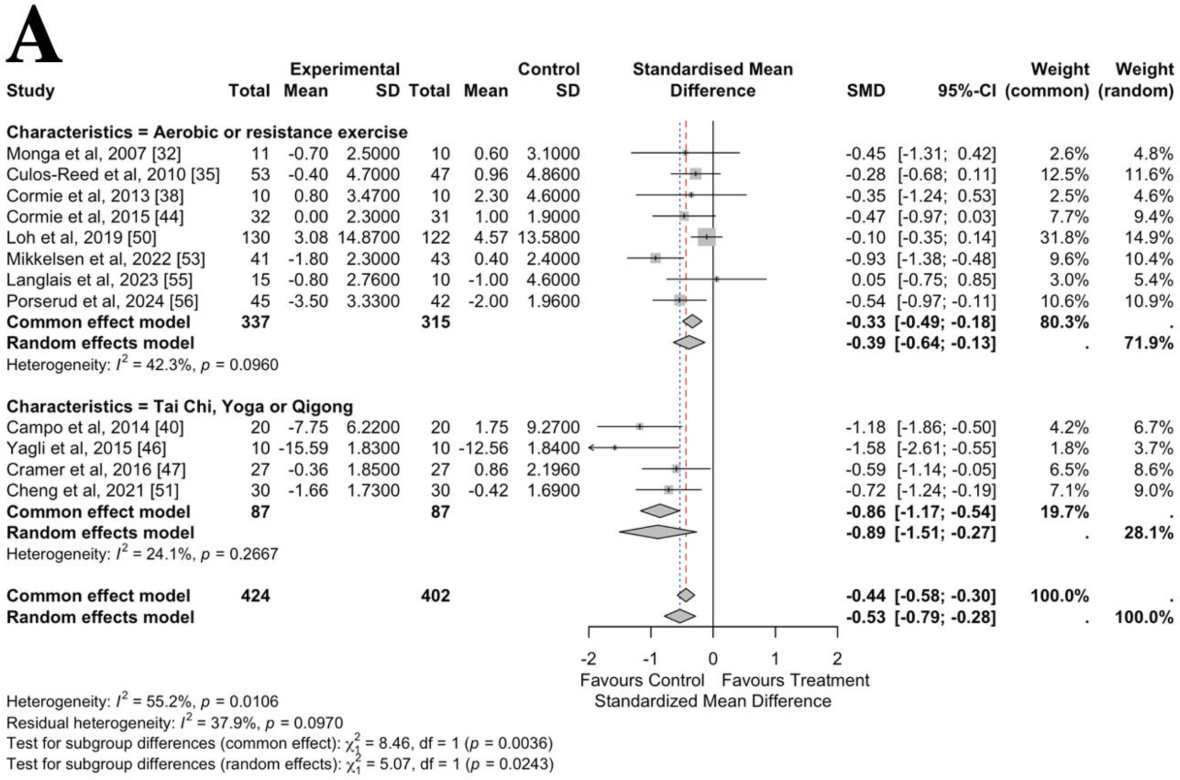

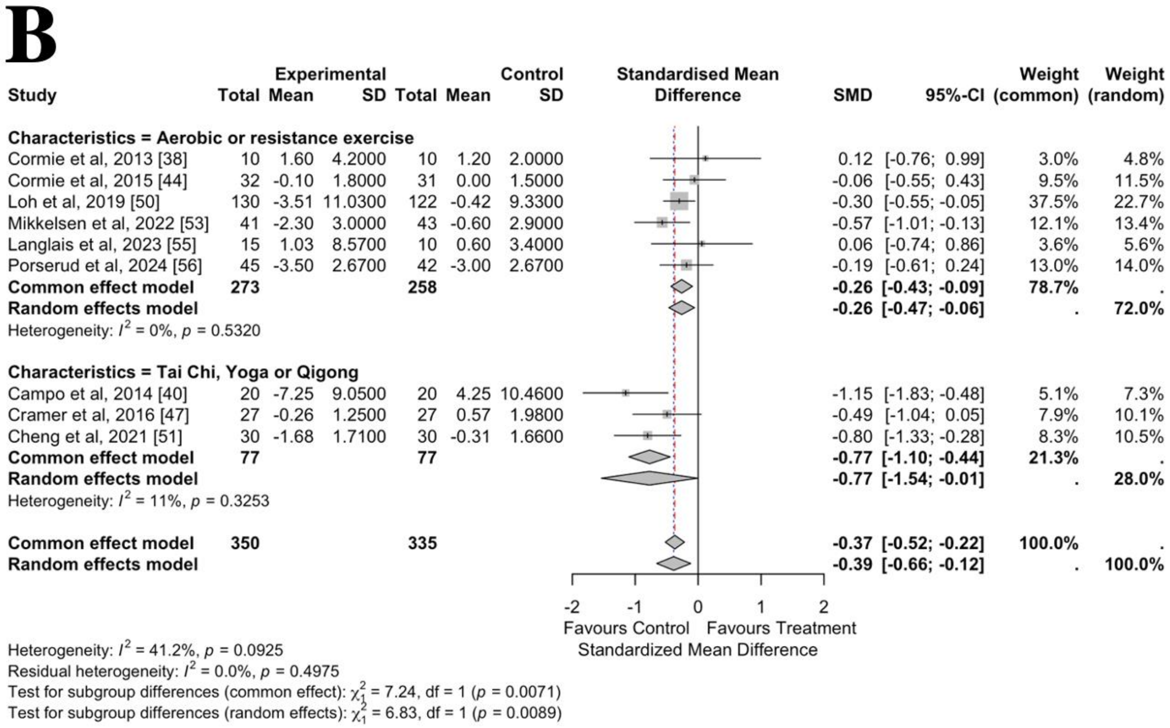

eFigure 1: Subgroup meta-analyses of exercise on depression (A) and anxiety (B) levels among older adults with cancer stratified by nature of exercise.

© 2025 Soong RY et al. *JAMA Netw. Open*.

eFigure 2: Funnel plot for visual inspection of publication bias in studies assessing depression severity in OAC

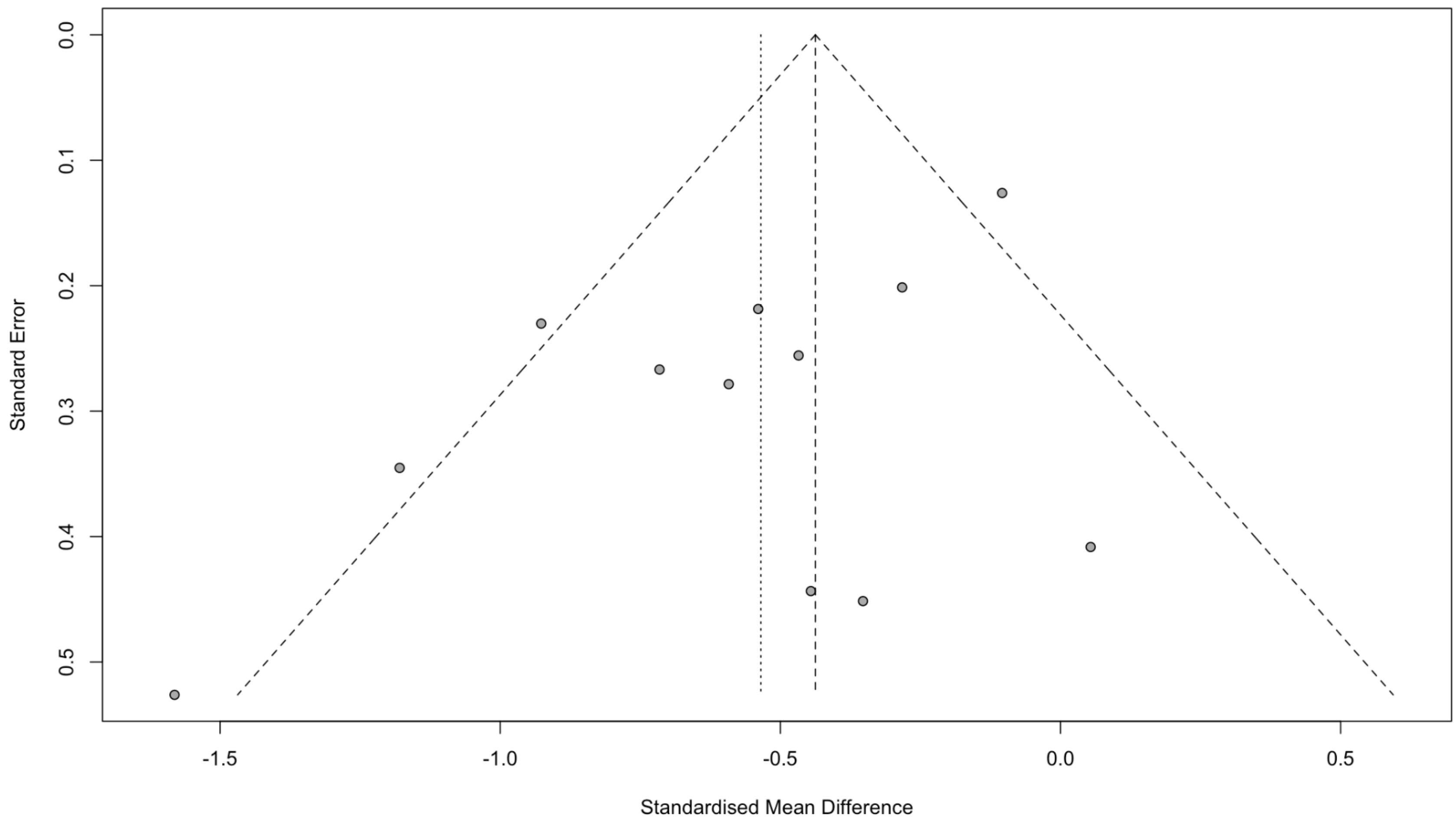

© 2025 Soong RY et al. *JAMA Netw. Open*.

eFigure 3: Trim-and-fill analysis for publication bias in studies assessing depression severity in OAC

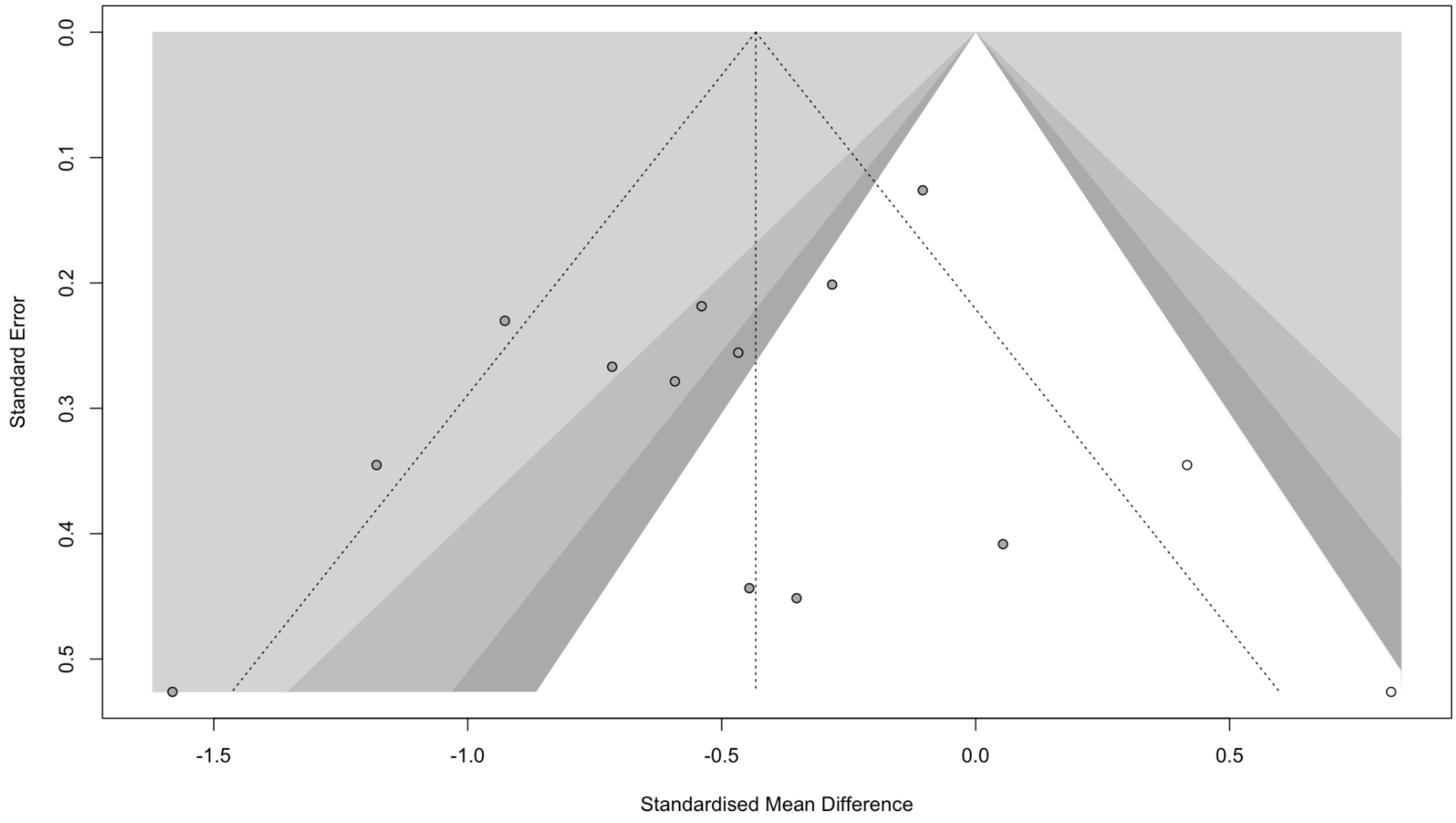

© 2025 Soong RY et al. *JAMA Netw. Open*.

eFigure 4: Quantitative assessment publication bias in studies assessing depression severity in OAC

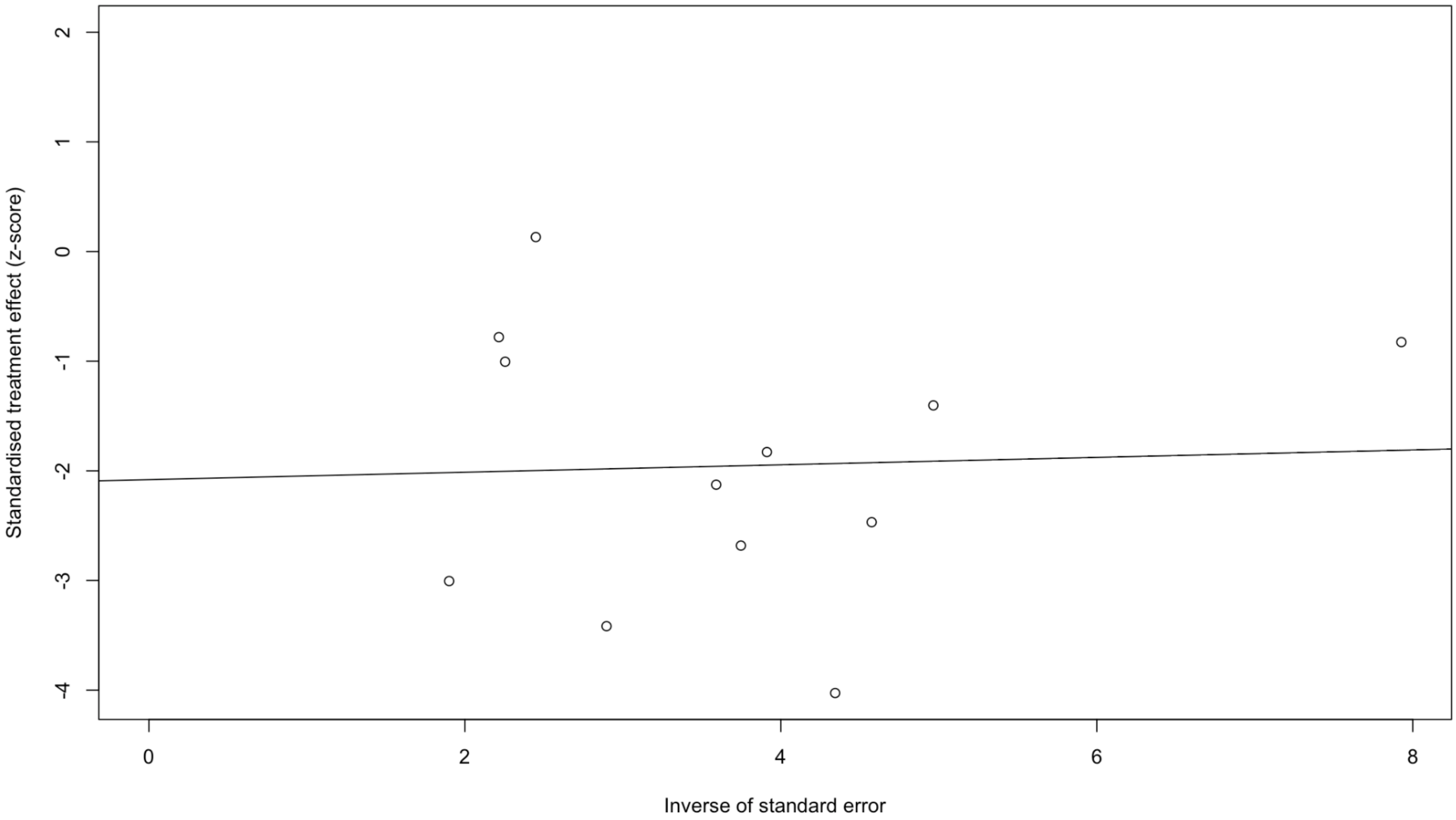

© 2025 Soong RY et al. *JAMA Netw. Open*.

eFigure 5: Leave-one-out analysis of studies assessing depression severity in OAC, using the random effects model

Study	SMD	95%-CI	P-value	Tau2	Tau	I2
Omitting Monga et al, 2007 [32]	-0.54	[-0.82; -0.27]	0.0013	0.0763	0.2763	59.3%
Omitting Culos-Reed et al, 2010 [35]	-0.57	[-0.85; -0.29]	0.0010	0.0778	0.2790	58.1%
Omitting Cormie et al, 2013 [38]	-0.55	[-0.82; -0.27]	0.0012	0.0760	0.2756	59.2%
Omitting Campo et al, 2014 [40]	-0.48	[-0.72; -0.24]	0.0013	0.0519	0.2277	49.3%
Omitting Cormie et al, 2015 [44]	-0.55	[-0.83; -0.27]	0.0015	0.0834	0.2888	59.2%
Omitting Yagli et al, 2015 [46]	-0.49	[-0.72; -0.26]	0.0007	0.0553	0.2353	49.3%
Omitting Cramer et al, 2016 [47]	-0.53	[-0.82; -0.25]	0.0017	0.0811	0.2849	58.7%
Omitting Loh et al, 2019 [50]	-0.60	[-0.84; -0.36]	0.0002	0.0234	0.1531	30.1%
Omitting Cheng et al, 2021 [51]	-0.52	[-0.80; -0.24]	0.0019	0.0769	0.2773	57.2%
Omitting Mikkelsen et al, 2022 [53]	-0.48	[-0.74; -0.22]	0.0021	0.0546	0.2336	48.9%
Omitting Langlais et al, 2023 [55]	-0.57	[-0.82; -0.31]	0.0006	0.0692	0.2630	56.6%
Omitting Porserud et al, 2024 [56]	-0.54	[-0.83; -0.26]	0.0017	0.0852	0.2919	58.9%
**Random effects model**	**-0.53**	**[-0.79; -0.28]**	**0.0007**	**0.0686**	**0.2618**	**55.2%**

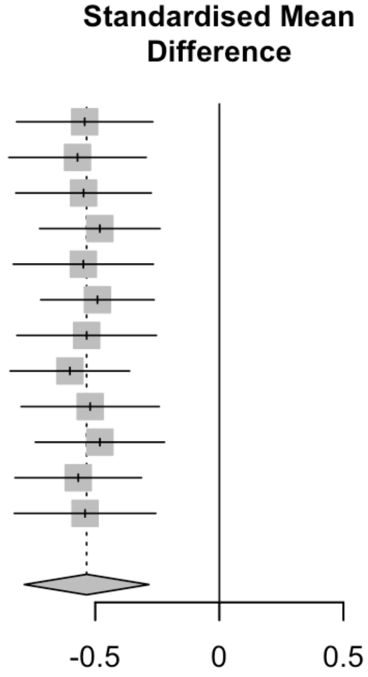

© 2025 Soong RY et al. *JAMA Netw. Open*.

eFigure 6: Outlier assessment of studies assessing depression severity in OAC, using the random effects model

Study	Experimental Total	Experimental Mean	Experimental SD	Control Total	Control Mean	Control SD	SMD	95%-CI	Weight
Monga et al, 2007 [32]	11	-0.70	2.5000	10	0.60	3.1000	-0.45	[-1.31; 0.42]	4.8%
Culos-Reed et al, 2010 [35]	53	-0.40	4.7000	47	0.96	4.8600	-0.28	[-0.68; 0.11]	11.6%
Cormie et al, 2013 [38]	10	0.80	3.4700	10	2.30	4.6000	-0.35	[-1.24; 0.53]	4.6%
Campo et al, 2014 [40]	20	-7.75	6.2200	20	1.75	9.2700	-1.18	[-1.86; -0.50]	6.7%
Cormie et al, 2015 [44]	32	0.00	2.3000	31	1.00	1.9000	-0.47	[-0.97; 0.03]	9.4%
Yagli et al, 2015 [46]	10	-15.59	1.8300	10	-12.56	1.8400	-1.58	[-2.61; -0.55]	3.7%
Cramer et al, 2016 [47]	27	-0.36	1.8500	27	0.86	2.1960	-0.59	[-1.14; -0.05]	8.6%
Loh et al, 2019 [50]	130	3.08	14.8700	122	4.57	13.5800	-0.10	[-0.35; 0.14]	14.9%
Cheng et al, 2021 [51]	30	-1.66	1.7300	30	-0.42	1.6900	-0.72	[-1.24; -0.19]	9.0%
Mikkelsen et al, 2022 [53]	41	-1.80	2.3000	43	0.40	2.4000	-0.93	[-1.38; -0.48]	10.4%
Langlais et al, 2023 [55]	15	-0.80	2.7600	10	-1.00	4.6000	0.05	[-0.75; 0.85]	5.4%
Porserud et al, 2024 [56]	45	-3.50	3.3300	42	-2.00	1.9600	-0.54	[-0.97; -0.11]	10.9%
**Random effects model**	**424**			**402**			**-0.53**	**[-0.79; -0.28]**	**100.0%**

Heterogeneity: $I^2 = 55.2\%$, $\tau^2 = 0.0686$, $p = 0.0106$

Standardised Mean Difference

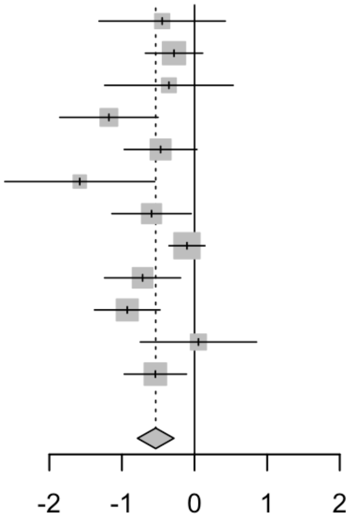

© 2025 Soong RY et al. *JAMA Netw. Open*.

eFigure 7: Funnel plot for visual inspection of publication bias in studies assessing anxiety severity in OAC

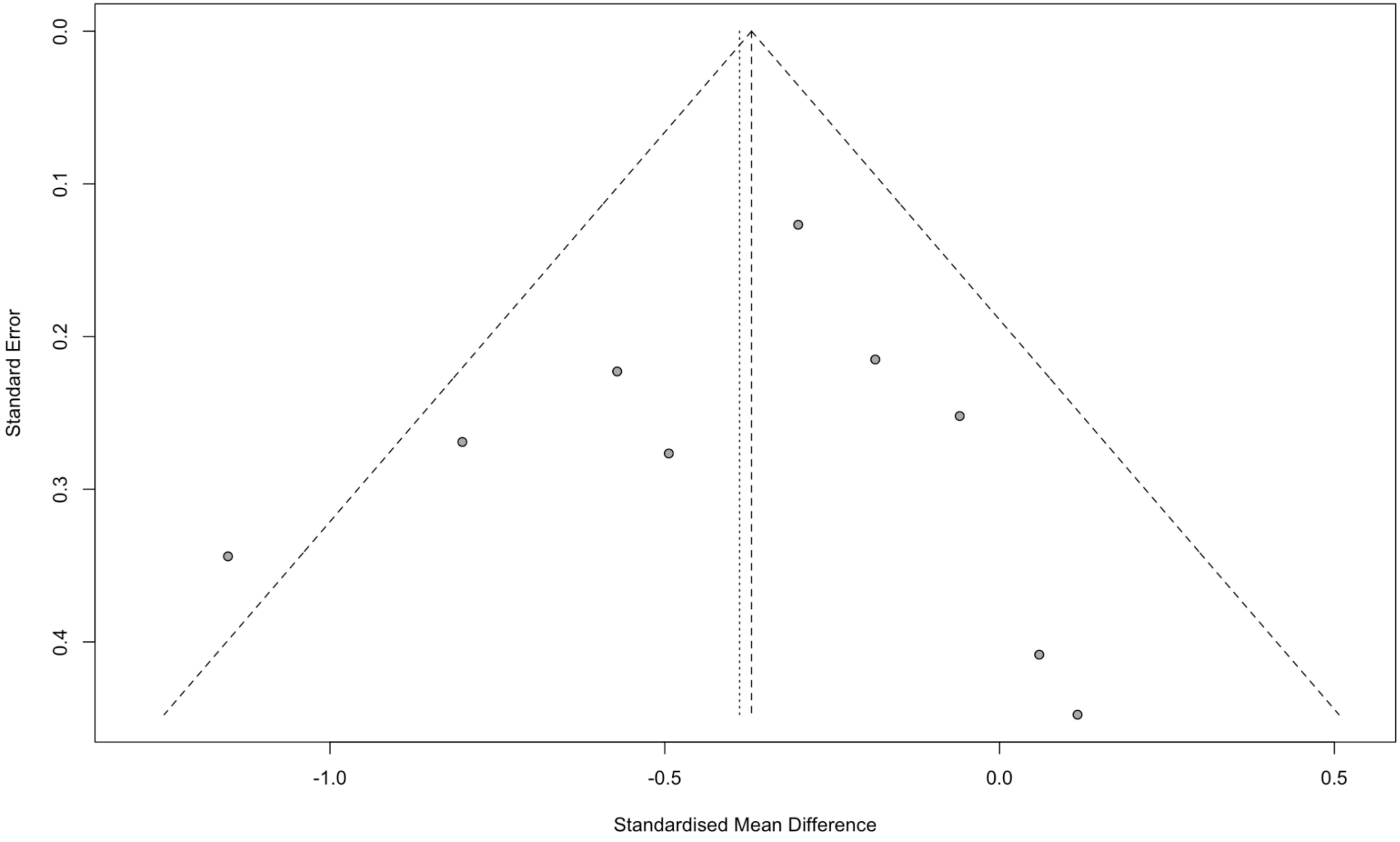

© 2025 Soong RY et al. *JAMA Netw. Open*.

eFigure 8: Trim-and-fill analysis for publication bias in studies assessing anxiety severity in OAC

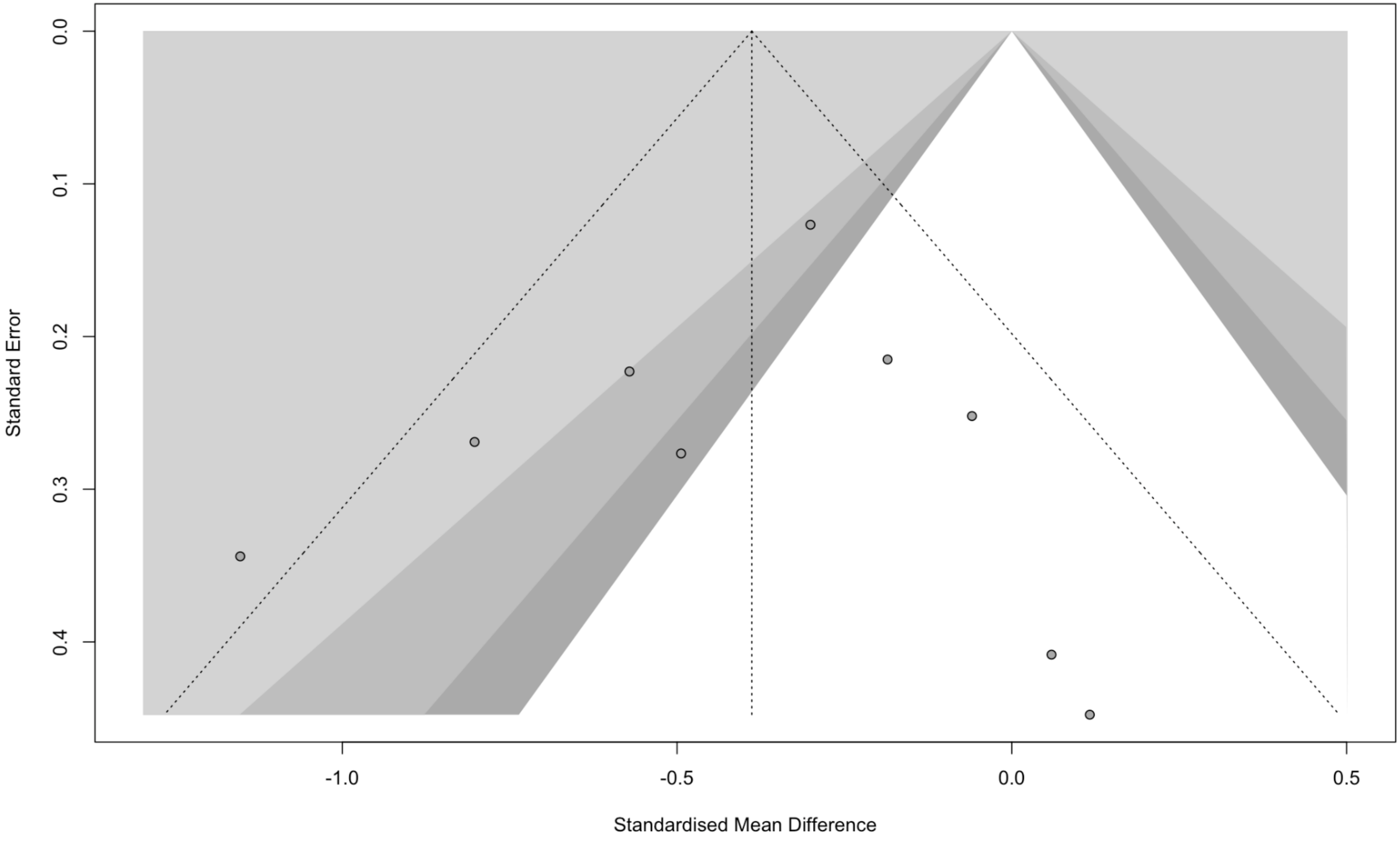

© 2025 Soong RY et al. *JAMA Netw. Open*.

eFigure 9: Quantitative assessment publication bias in studies assessing anxiety severity in OAC

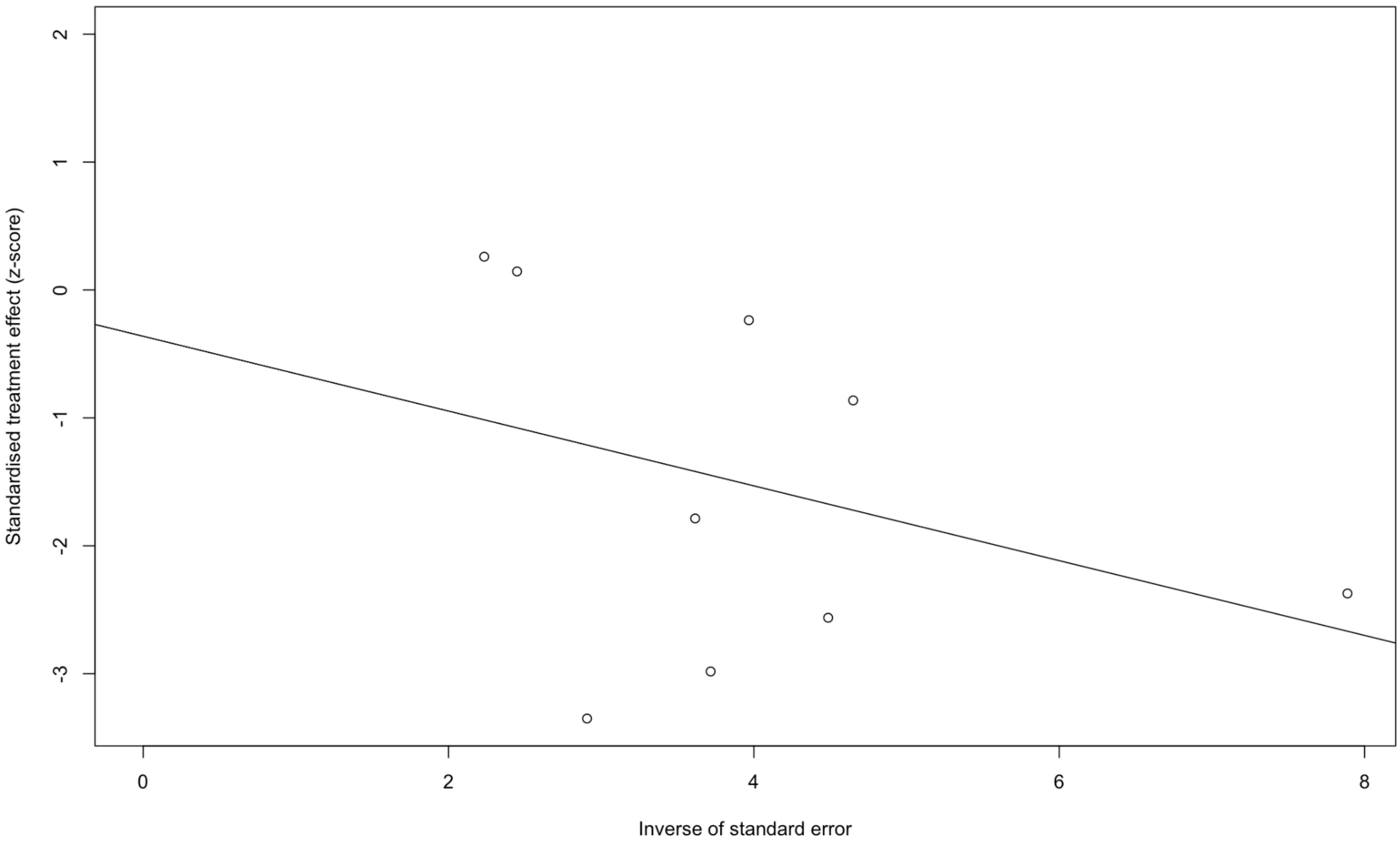

© 2025 Soong RY et al. *JAMA Netw. Open*.

eFigure 10: Leave-one-out analysis of studies assessing anxiety severity in OAC, using the random effects model

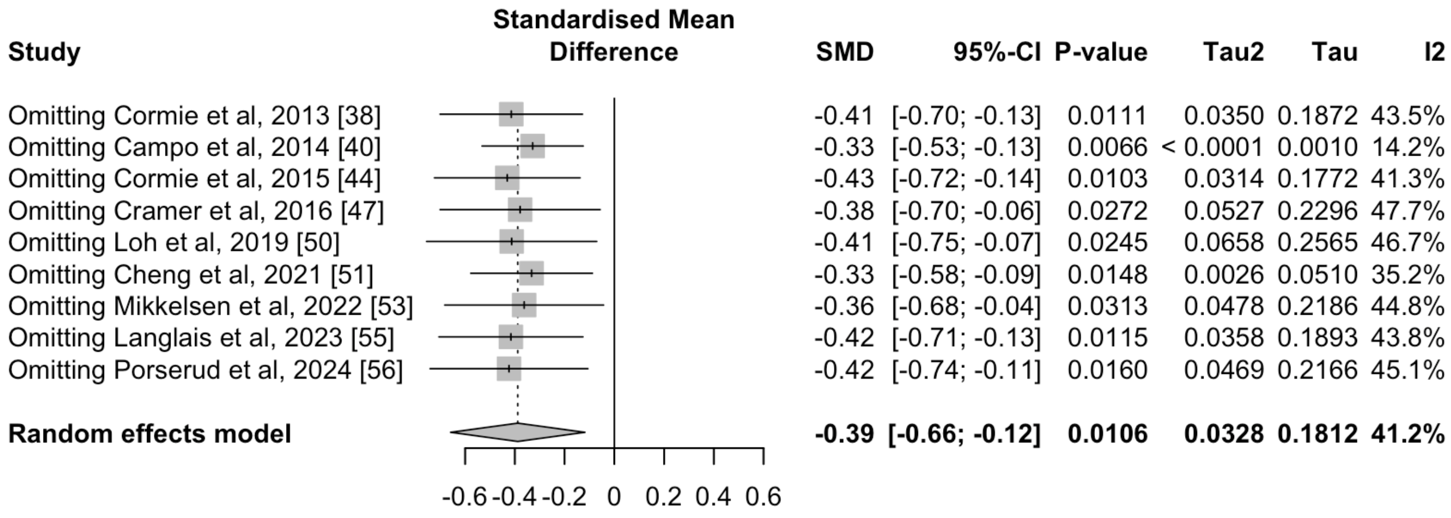

© 2025 Soong RY et al. *JAMA Netw. Open*.

eFigure 11: Outlier assessment of studies assessing anxiety severity in OAC, using the random effects model

Study	Experimental Total	Experimental Mean	Experimental SD	Control Total	Control Mean	Control SD	SMD	95%-CI	Weight
Cormie et al, 2013 [38]	10	1.60	4.2000	10	1.20	2.0000	0.12	[-0.76; 0.99]	4.8%
Campo et al, 2014 [40]	20	-7.25	9.0500	20	4.25	10.4600	-1.15	[-1.83; -0.48]	7.3%
Cormie et al, 2015 [44]	32	-0.10	1.8000	31	0.00	1.5000	-0.06	[-0.55; 0.43]	11.5%
Cramer et al, 2016 [47]	27	-0.26	1.2500	27	0.57	1.9800	-0.49	[-1.04; 0.05]	10.1%
Loh et al, 2019 [50]	130	-3.51	11.0300	122	-0.42	9.3300	-0.30	[-0.55; -0.05]	22.7%
Cheng et al, 2021 [51]	30	-1.68	1.7100	30	-0.31	1.6600	-0.80	[-1.33; -0.28]	10.5%
Mikkelsen et al, 2022 [53]	41	-2.30	3.0000	43	-0.60	2.9000	-0.57	[-1.01; -0.13]	13.4%
Langlais et al, 2023 [55]	15	1.03	8.5700	10	0.60	3.4000	0.06	[-0.74; 0.86]	5.6%
Porserud et al, 2024 [56]	45	-3.50	2.6700	42	-3.00	2.6700	-0.19	[-0.61; 0.24]	14.0%
**Random effects model**	**350**			**335**			**-0.39**	**[-0.66; -0.12]**	**100.0%**

Heterogeneity: $I^2 = 41.2\%$, $\tau^2 = 0.0328$, $p = 0.0925$

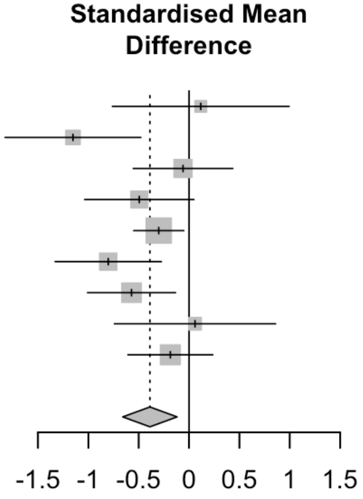

© 2025 Soong RY et al. *JAMA Netw. Open*.

eFigure 12: Funnel plot for visual inspection of publication bias in studies assessing QoL levels in OAC

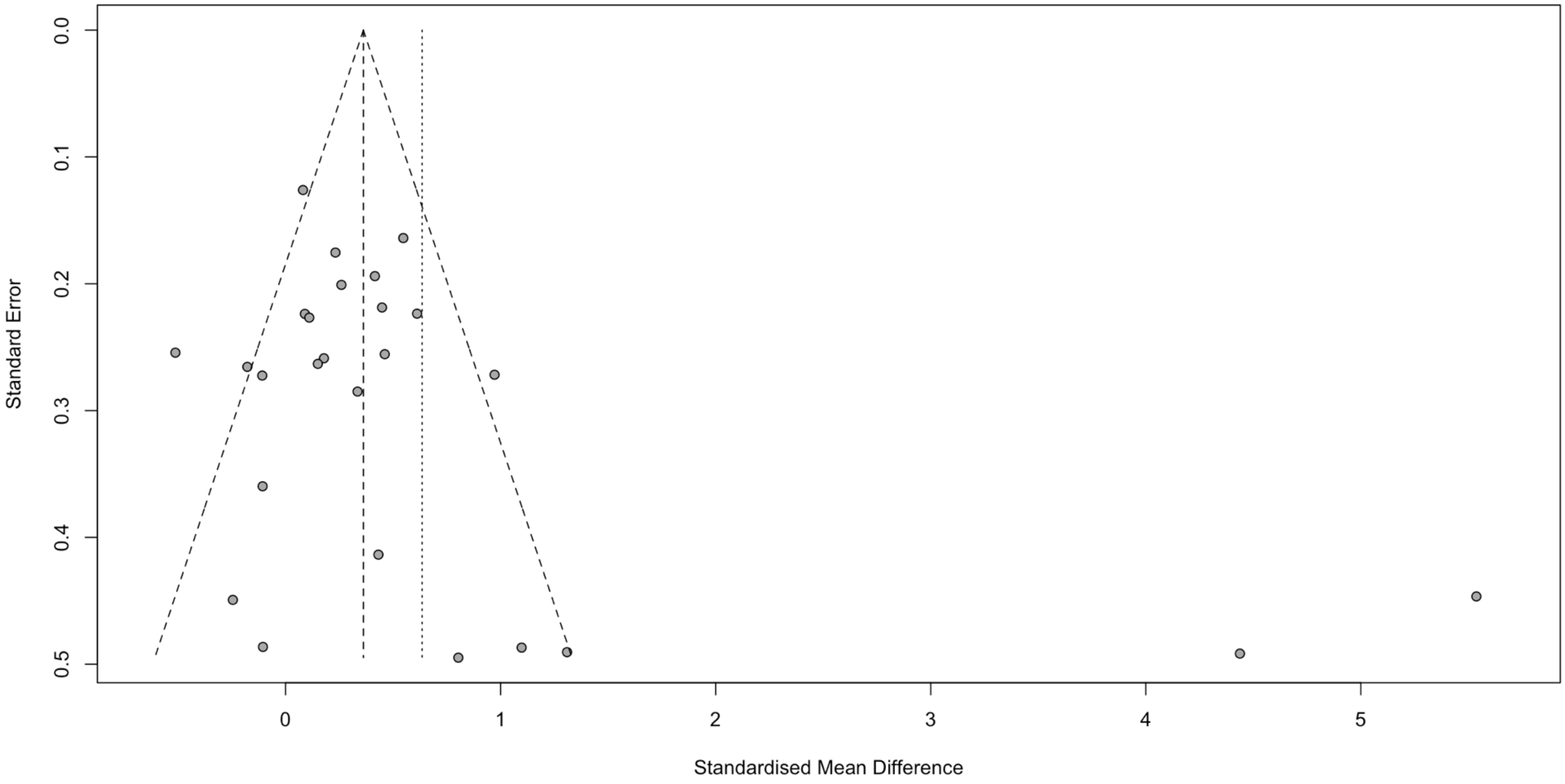

© 2025 Soong RY et al. *JAMA Netw. Open*.

eFigure 13: Trim-and-fill analysis for publication bias in studies assessing QoL levels in OAC

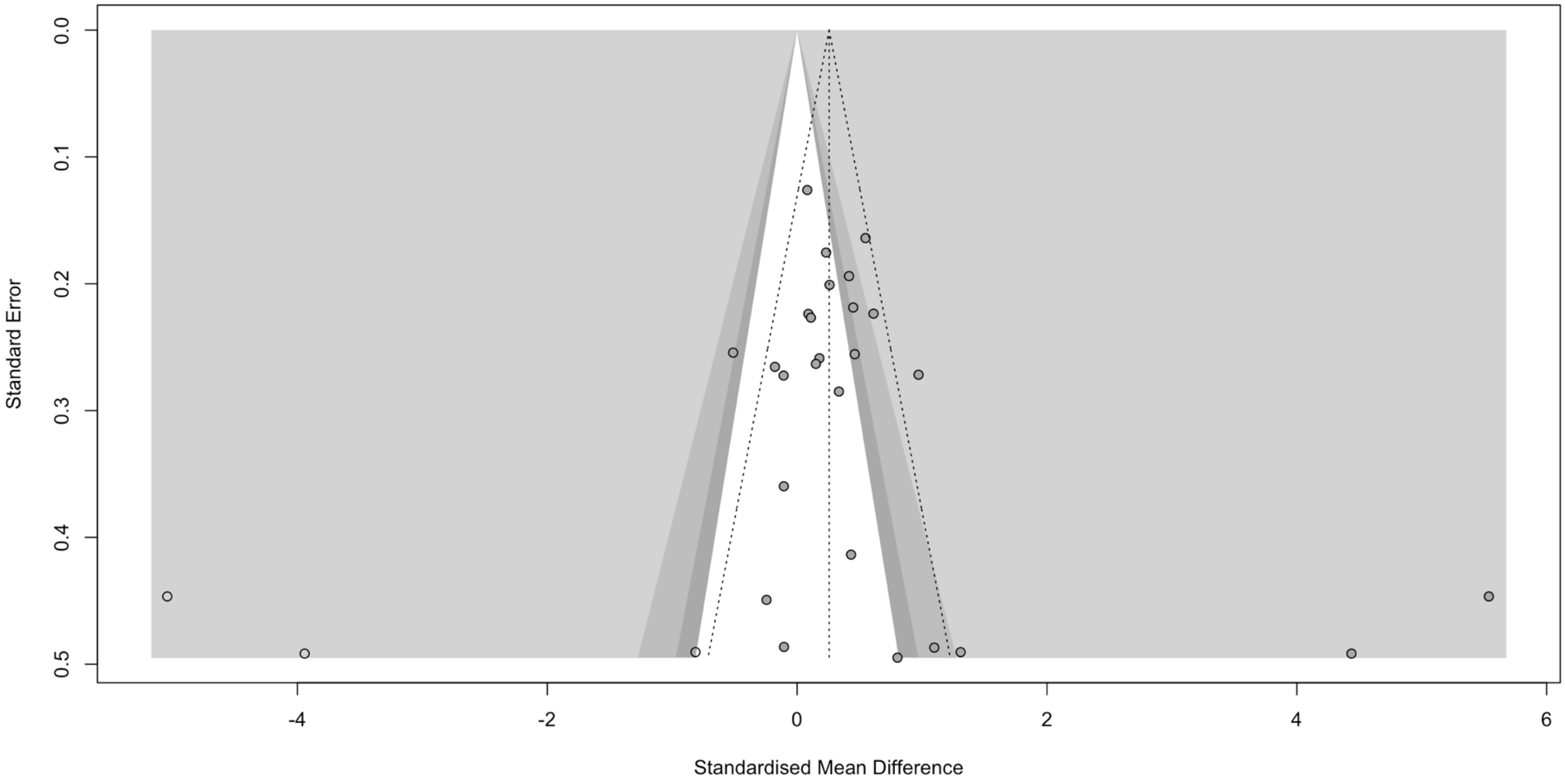

© 2025 Soong RY et al. *JAMA Netw. Open*.

eFigure 14: Quantitative assessment publication bias in studies assessing QoL levels in OAC

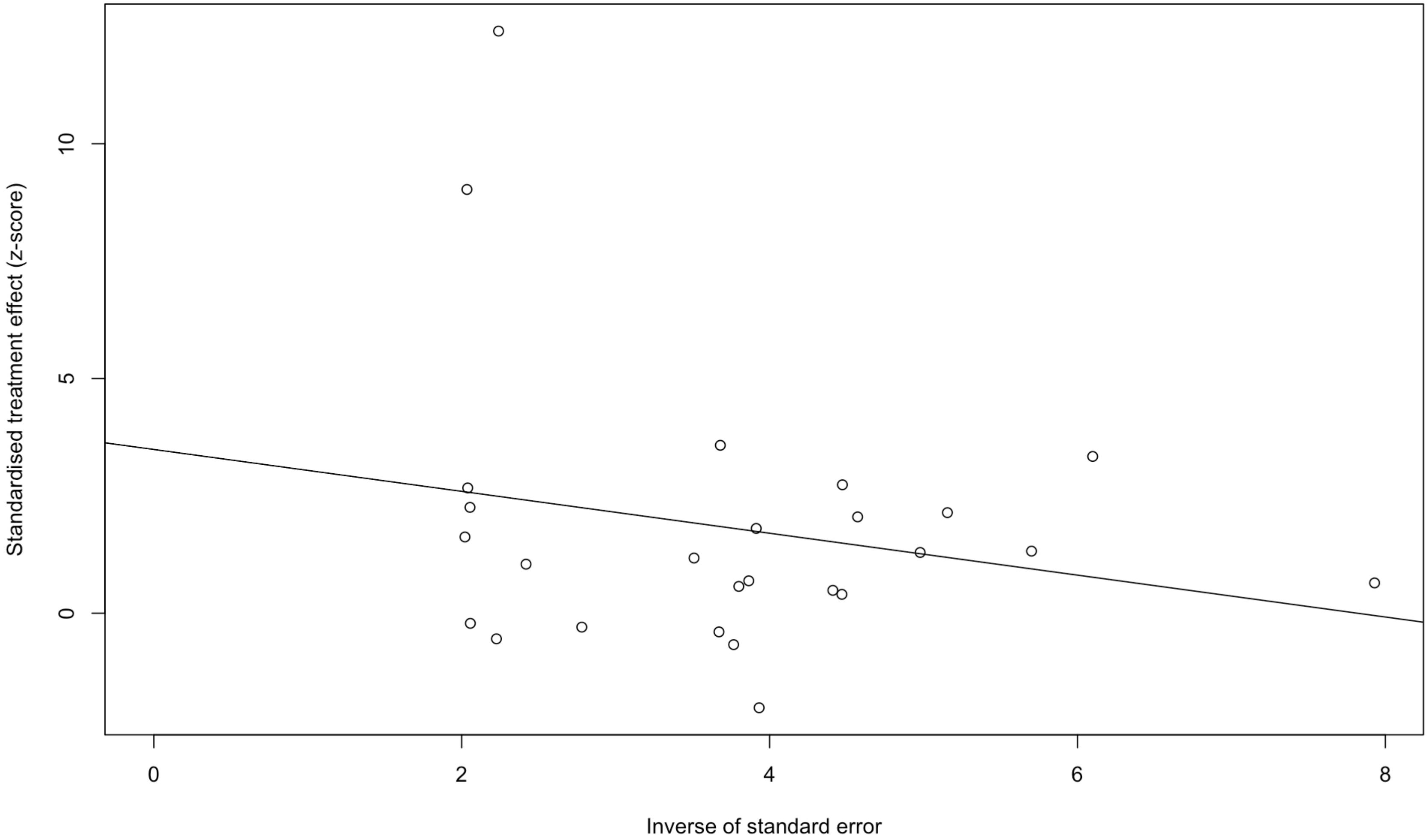

© 2025 Soong RY et al. *JAMA Netw. Open*.

eFigure 15: Leave-one-out analysis of studies assessing QoL levels in OAC, using the random effects model

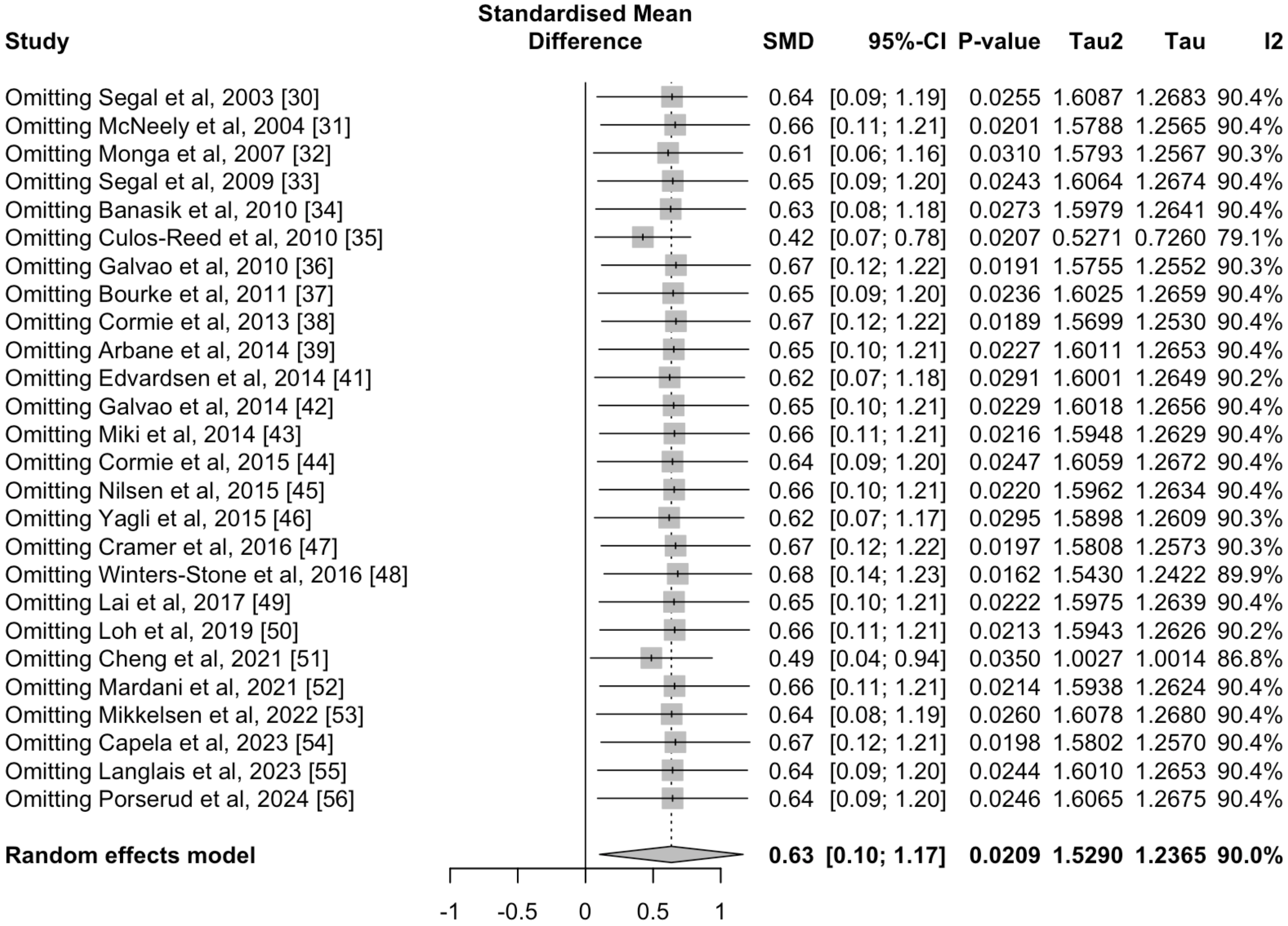

© 2025 Soong RY et al. *JAMA Netw. Open*.

eFigure 16: Outlier assessment of studies assessing QoL levels in OAC, using the random effects model

Study	Experimental Total	Experimental Mean	Experimental SD	Control Total	Control Mean	Control SD	SMD	95%-CI	Weight
Segal et al, 2003 [30]	82	2.00	9.1000	73	-3.30	10.2000	0.55	[0.23; 0.87]	7.8%
McNeely et al, 2004 [31]	8	-0.50	7.9000	9	0.68	12.6000	-0.10	[-1.06; 0.85]	1.5%
Monga et al, 2007 [32]	11	7.40	10.4000	10	-6.40	9.8000	1.31	[0.35; 2.27]	1.5%
Segal et al, 2009 [33]	80	3.25	8.2500	41	-0.20	8.2500	0.42	[0.04; 0.80]	6.4%
Banasik et al, 2010 [34]	9	0.32	0.2300	9	0.08	0.3300	0.80	[-0.17; 1.77]	1.4%
Culos-Reed et al, 2010 [35]	53	2.70	0.9200	47	-2.33	0.8800	5.54	[4.66; 6.41]	0.0%
Galvao et al, 2010 [36]	29	-0.60	6.2400	28	0.60	7.0700	-0.18	[-0.70; 0.34]	4.2%
Bourke et al, 2011 [37]	25	0.00	6.0800	25	-3.00	10.8900	0.33	[-0.22; 0.89]	3.7%
Cormie et al, 2013 [38]	10	-1.50	7.7400	10	0.40	7.1000	-0.24	[-1.13; 0.64]	1.7%
Arbane et al, 2014 [39]	64	-2.00	51.0400	67	-15.00	59.8600	0.23	[-0.11; 0.58]	7.2%
Edvardsen et al, 2014 [41]	30	9.10	7.5600	31	1.20	8.4500	0.97	[0.44; 1.50]	4.0%
Galvao et al, 2014 [42]	50	1.30	5.8600	50	-0.30	6.3500	0.26	[-0.13; 0.65]	6.1%
Miki et al, 2014 [43]	38	2.18	9.5600	40	1.12	9.4500	0.11	[-0.33; 0.55]	5.2%
Cormie et al, 2015 [44]	32	1.90	6.8000	31	-1.30	6.9000	0.46	[-0.04; 0.96]	4.4%
Nilsen et al, 2015 [45]	28	1.50	12.2500	30	0.00	6.9100	0.15	[-0.37; 0.67]	4.2%
Yagli et al, 2015 [46]	10	239.63	61.5600	10	169.44	60.9000	1.10	[0.14; 2.05]	1.5%
Cramer et al, 2016 [47]	27	-1.32	9.0100	27	-0.21	11.0800	-0.11	[-0.64; 0.43]	4.0%
Winters-Stone et al, 2016 [48]	32	-1.10	5.4200	32	1.70	5.3800	-0.51	[-1.01; -0.01]	0.0%
Lai et al, 2017 [49]	30	1.60	8.7000	30	0.10	7.8600	0.18	[-0.33; 0.69]	4.3%
Loh et al, 2019 [50]	130	1.04	2.8900	122	0.80	3.0000	0.08	[-0.17; 0.33]	10.0%
Cheng et al, 2021 [51]	30	6.70	4.1600	30	-8.60	2.4200	4.44	[3.47; 5.40]	0.0%
Mardani et al, 2021 [52]	40	-0.71	8.1100	40	-1.39	6.8300	0.09	[-0.35; 0.53]	5.3%
Mikkelsen et al, 2022 [53]	41	9.80	22.4000	43	-3.20	19.7000	0.61	[0.17; 1.05]	5.3%
Capela et al, 2023 [54]	16	-4.16	22.5000	15	-1.40	27.8200	-0.11	[-0.81; 0.60]	2.5%
Langlais et al, 2023 [55]	15	1.91	2.3100	10	0.60	3.7000	0.43	[-0.38; 1.24]	2.0%
Porserud et al, 2024 [56]	45	41.00	15.4600	41	34.00	15.4600	0.45	[0.02; 0.88]	5.5%
**Random effects model**	**965**			**901**			**0.30**	**[0.16; 0.44]**	**100.0%**

Heterogeneity: $I^2 = 34.9\%$, $\tau^2 = 0.0228$, $p = 0.0513$

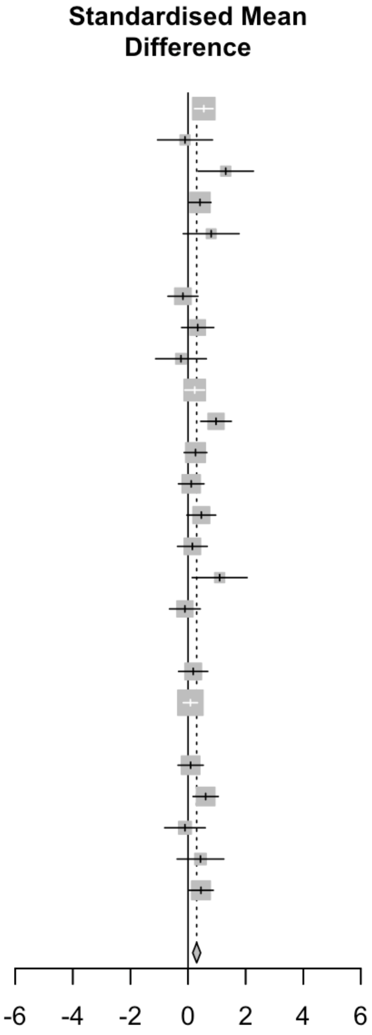

© 2025 Soong RY et al. *JAMA Netw. Open*.
