# Supplementary material for: Exercise Interventions for Depression, Anxiety, and Quality of Life in Older Adults With Cancer: A Systematic Review and Meta-Analysis
Source: JAMA Netw Open. 2025 Feb 4;8(2):e2457859. doi: 10.1001/jamanetworkopen.2024.57859 (PMC11795328; doi:10.1001/jamanetworkopen.2024.57859)
Supplement: Supplement 2. — Data Sharing Statement [file jamanetwopen-e2457859-s002.pdf]

Data Sharing Statement

Soong. Exercise Interventions for Depression, Anxiety, and Quality of Life in Older Adults With Cancer. *JAMA Netw Open*. Published February 04, 2025.
doi:10.1001/jamanetworkopen.2024.57859

Data
Data available: No
